# The Intratumoral Bacterial Metataxonomic Signature of Hepatocellular Carcinoma

**DOI:** 10.1128/spectrum.00983-22

**Published:** 2022-09-29

**Authors:** Jian-Hang Huang, Jie Wang, Xiao-Qiang Chai, Zhong-Chen Li, Ying-Hua Jiang, Jun Li, Xing Liu, Jia Fan, Jia-Bin Cai, Feng Liu

**Affiliations:** a Minhang Hospital, Fudan University, and Shanghai Key Laboratory of Medical Epigenetics, International Co-laboratory of Medical Epigenetics and Metabolism, Ministry of Science and Technology, Institutes of Biomedical of Sciences, Fudan University, Shanghai, China; b Department of Liver Surgery and Transplantation of Zhongshan Hospital, Liver Cancer Institute of Zhongshan Hospital, Key Laboratory of Carcinogenesis and Cancer Invasion of Ministry of Education, Fudan University, Shanghai, China; c Department of General Surgery, Shanghai TongRen Hospital, Shanghai, China; d Department of Central Laboratory Medicine, Shanghai Municipal Hospital of Traditional Chinese Medicine, Shanghai University of Traditional Chinese Medicine, Shanghai, China; Wayne State University

**Keywords:** hepatocellular carcinoma, microbiota, fluorescence *in situ* hybridization, bacteria, machine learning

## Abstract

Microbiota is implicated in hepatocellular carcinoma (HCC). The spectrum of intratumoral microbiota associated with HCC progression remains elusive. Fluorescence *in situ* hybridization revealed that microbial DNAs were distributed in the cytosol of liver hepatocytes and erythrocytes. Viable anaerobic or aerobic bacteria were recovered in HCC tissues by fresh tissue culture. We performed a comprehensive DNA sequencing of bacterial 16S rRNA genes in 156 samples from 28 normal liver, 64 peritumoral, and 64 HCC tissues, and the DNA sequencing yielded 4.2 million high-quality reads. Both alpha and beta diversity in peritumor and HCC microbiota were increased compared to normal controls. The most predominant phyla in HCC were *Patescibacteria*, *Proteobacteria*, *Bacteroidota*, *Firmicutes*, and *Actinobacteriota*. phyla of *Proteobacteria*, *Firmicutes*, and *Actinobacteriota*, and classes of *Bacilli* and *Actinobacteria*, were consistently enriched in peritumor and HCC tissues, while *Gammaproteobacteria* was especially abundant in HCC tissues compared to normal controls. *Streptococcaceae* and *Lactococcus* were the marker taxa of HCC cirrhosis. The Staphylococcus branch and *Caulobacter* branch were selectively enriched in HBV-negative HCCs. The abundance of *Proteobacteria*, *Gammaproteobacteria*, *Firmicutes*, *Actinobacteriota*, and *Saccharimonadia* were associated with the clinicopathological features of HCC patients. The inferred functions of different taxa were changed between the microbiota of normal liver and peritumor/HCC. Random forest machine learning achieved great discriminative performance in HCC prediction (area under the curve [AUC] = 1.00 in the training cohort, AUC = 0.950 for top five class signature, and AUC = 0.943 for the top 50 operational taxonomy units [OTUs] in the validation cohort). Our analysis highlights the complexity and diversity of the liver and HCC microbiota and established a specific intratumoral microbial signature for the potential prediction of HCC.

**IMPORTANCE** Gut microbiome is an important regulator of hepatic inflammation, detoxification, and immunity, and contributes to the carcinogenesis of liver cancer. Intratumoral bacteria are supposed to be closer to the tumor cells, forming a microenvironment that may be relevant to the pathological process of hepatocellular carcinoma (HCC). However, the presence of viable intratumoral bacteria remains unclear. It is worth exploring whether the metataxonomic characteristics of intratumoral bacteria can be used as a potential marker for HCC prediction. Here, we present the first evidence of the existence of viable intratumoral bacteria in HCC using the tissue culture method. We revealed that microbial DNAs were distributed in the cytosol of liver hepatocytes and erythrocytes. We analyzed the diversity, structure, and abundance of normal liver and HCC microbiota. We built a machine learning model for HCC prediction using intratumoral bacterial features. We show that specific taxa represent potential targets for both therapeutic and diagnostic interventions.

## INTRODUCTION

Liver cancer is the sixth most common cancer and the fourth most deadly cancer worldwide (1). According to GLOBALCAN statistics, there were 840,000 new cases and 780,000 liver cancer deaths in 2018 (2). Hepatocellular carcinoma (HCC) accounts for 75% to 85% of primary liver cancer cases, which may be caused by alcoholic hepatitis, nonalcoholic steatohepatitis, obesity, smoking, type 2 diabetes, or aflatoxin exposure (2). However, the major risk factor of HCC (especially in Asia) is chronic infection with hepatitis B virus (HBV) or hepatitis C virus (HCV) (3).

Other microorganisms may also lead to the occurrence and development of HCC and other liver diseases. Through the gut-liver axis, gut microbiota may modulate liver diseases, including liver cancer, by transporting bacteria and their metabolites to the liver through the vascular and portal circulation systems (4, 5). Gut microbiota can influence the hepatotoxicity of certain drugs, such as tacrine (6). Intestinal microbiota and bacteria-derived lipopolysaccharide and Toll-like receptors have been shown to promote HCC (7). Gut bacteria mediate the conversion of primary bile acid to secondary bile acid, increase the accumulation of natural killer (NK) and T cells and stimulate the production of interferon gamma, thereby eliciting an antitumor effect in HCC (8). Gut microbial metabolites, such as lipoteichoic acid and deoxycholic acid, contribute to the development of HCC (9, 10). These data suggest that commensal bacteria are important regulators of hepatic inflammation, detoxification, and immunity, and contribute to chronic diseases, fibrosis, and liver cancer. A comprehensive metataxonomic analysis has been performed to address the composition, abundance, and dysbiosis of gut microbiota of liver diseases, including HCC (5, 11). The gut microbiome of patients with liver cirrhosis was characterized and compared with that of healthy controls. The results showed that most of the gut species enriched in patients with liver cirrhosis originated from the oral cavity (12).

In addition to the gut microbiota, tissue-enriched and intratumoral bacteria may be associated with carcinogenesis. There is growing evidence that different types of human cancers, such as breast, lung, ovary, pancreas, melanoma, bone, and brain tumors, have a unique microbiota composition of intratumoral bacteria (13). Interestingly, most of the intratumoral bacteria in these cancer types are located intracellularly and are found in both cancer cells and immune cells (13). The analysis of the microbiota of pancreatic adenocarcinoma showed different alpha-diversity in patients with long-term or short-term survival, with the tumor microbiota cross-talking with the gut microbiota and modulating the host’s immune response (14). These findings indicate that the intratumoral microbiota is heterogeneous across different cancer types and may also differ from the associated gut microbiota. The intratumoral microbiota may contribute to cancer carcinogenesis or immunity through different mechanisms and signaling. In contrast to the gut microbiota, the intratumoral microbiota of liver cancer remains elusive. Although a recent study has shown that the liver tissue in nonalcoholic fatty liver disease (NAFLD) has a taxonomic profile of a variety of bacteria DNA (15), the presence of viable bacteria in the liver and the cellular distribution of intratumoral bacteria remain known. Furthermore, the dysbiosis of the HCC microbiota, the disease state-specific taxa of HCC, and the association of the bacteria burden with the clinicopathological characteristics of HCC should be addressed.

Here, we analyzed the microbial spectrum of human normal liver, peritumor, and HCC tissues using 16S rRNA gene sequencing of 155 liver samples and identified specific microbial signatures that could be potentially used for HCC diagnosis. We also verified the existence of intratumoral microbes in HCC using fluorescence *in situ* hybridization (FISH) and fresh tissue culture methods.

## RESULTS

### HCC microbial DNA was enriched in the cytoplasm of hepatocytes and erythrocytes.

**FIG 1:**
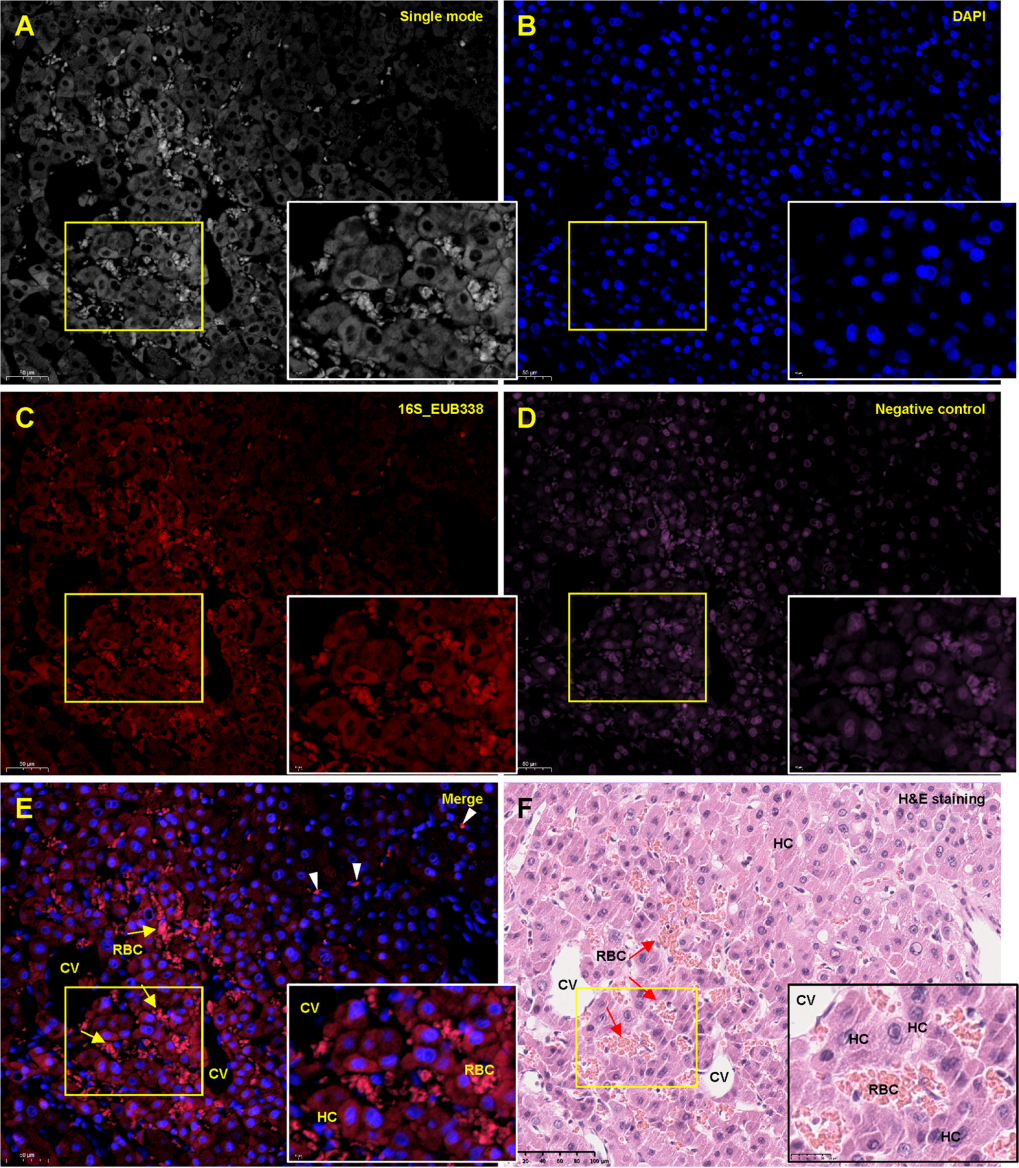
FISH analysis of liver cancer. Formalin-fixed paraffin-embedded (FFPE) liver cancer sections were deparaffinized, rehydrated, and probed with Cy3-labeled probes EUB338 (5′-GCTGCCTCCCGTAGGAGT-3′) (red) or Cy5-labeled nonspecific complement probe (5′-CGACGGAGGGCATCCTCA-3′) (pink). Both probes have been recently used to analyze the intratumoral microbiota of human cancer tissues (13). The nuclei were counterstained with diamidino-phenyl-indole (DAPI). A. Single-mode image of the staining. The yellow box indicates the region enlarged in the low right. B. DAPI staining of the nuclei. C. EUB338 staining. D. Negative staining. E. The merged image of different staining. The yellow arrows indicate clustered RBCs in the congestion area. The arrowheads indicate RBCs outside the congestion area. F. Hematoxylin-eosin staining of a sequential tissue section. The region corresponding to the FISH image was shown. RBC, red blood cell. HC, hepatocyte. CV, central vein.

To evaluate the distribution and abundance of the microbial DNA in liver cancer, we performed a FISH analysis of an HCC tissue and its related peritumor tissue freshly collected during cancer resection, using a 16S rRNA probe, as previously reported (13). For comparison purposes, we repeated hematoxylin and eosin (H&E) staining and Gram staining with consecutive sections (see Fig. S1A in the supplemental material). The FISH signals were distributed in the cancerous and peritumoral tissues and concentrated in some areas (Fig. S1B). Hepatocytes (HCC tissue) within the congestion area were also strongly stained, especially in the cytosol rather than the nucleus (Fig. 1). In the peritumoral tissue, we found that the FISH signals were enriched in the nucleus-free red blood cells (RBCs) in several expanded sinusoid areas that emitted the strongest signals (Fig. S2). The accumulation of RBCs in the sinusoid indicated serious congestion of the injured liver tissue. Negative probe staining also showed visible signal, but mainly concentrated on the nuclei. Hepatic bile ductules circulated with simple columnar epithelium were negative for bacterial 16S rRNA probe staining (Fig. S3 and S4).

### Viable and infectious bacteria were present in HCC tissues.

To investigate the presence of viable bacteria in the liver, we performed tissue culture using fresh HCC tissues collected during surgery. As a positive control, the aerobic cultures of an environment sample yielded thousands of colonies, while the anaerobic cultures produced more than 30 visible colonies (Fig. S5A and B). On the other hand, aerobic or anaerobic cultures of negative controls yielded no visible colonies even after prolonged incubation, indicating the reliability of our tissue culture method (Fig. S5C and D). Twelve paired peritumoral and HCC tissues were homogenized and subjected to aerobic and anaerobic cultures in parallel (Table S1). After prolonged incubation of the liver samples, two peritumor tissues and three HCC tissues grew visible colonies, while no colonies appeared in the cultures of the other samples (Fig. S5E–J). These colonies were subjected to colony PCR (using 16S rRNA gene primers) and DNA sequencing (Table S2A). Thirteen colonies were identified, of which two colonies (14P-G-2 and 9T-G-1) were Staphylococcus aureus (Fig. S5G and H), while eight colonies were facultative anaerobes of the genus *Rothia*. Two colonies in the HCC tissues were aerobic species of the family *Bacillaceae* (1T-B-1 and 1T-B-2) (Fig. S5I). One colony was a microaerophilic bacterium belonging to the genus *Corynebacterium* sp. (Fig. S5J). These results suggest that the liver cancer tissue contains viable intratumoral bacteria.

### The metataxonomic analysis of liver cancer.

16S rRNA gene sequencing represents a robust and culture-independent strategy for determining the structure and abundance of environmental microbiota. We performed high-throughput 16S rRNA gene sequencing to address the structure and abundance of the liver cancer microbiota. The “16S_rRNA cohort” contains 168 liver tissue samples from 100 individuals, including 68 HCCs and paired peritumor tissues, 3 independent peritumor tissues, and 29 normal liver tissues which were isolated from patients with liver metastasis of colon cancer or other non-HCC diseases (Fig. S6A, Table S1). The median age of the control and HCC groups was comparable (58 versus 60 years), while the incidence was higher in men with HCC (56 men versus 15 women) compared to controls (13 men versus 16 women).

The 16S rRNA genes of 155 samples were successfully amplified and sequenced, while 13 samples failed with no visible PCR bands (Fig. S6A). We also sequenced 14 negative-control samples, including six DNA extraction controls that used blank extraction reagents in two extraction experiments and eight PCR amplification controls that used sterile water in three batches of PCR experiments. The concentrations of the DNA extracts and the PCR product were extremely lower in the extraction negative controls than that of the liver tissue samples (Fig. S6B). The sequencing yielded 4,191,508 high-quality and valid reads for liver samples, and 23,854 valid reads for the blank reagent controls (Table S2B). Seven liver tissues with valid reads <10,000 were considered unsuccessful and excluded from downstream analysis (Fig. S6B). The negative control results were used for contamination filtering by the decontam program (16). Finally, the sequences were assembled into 3,504 operational taxonomy units (OTUs) with 97% sequence similarity and taxonomic identities were determined.

### Alpha diversity of the microbiota was significantly higher in HCC than in the normal liver.

**FIG 2:**
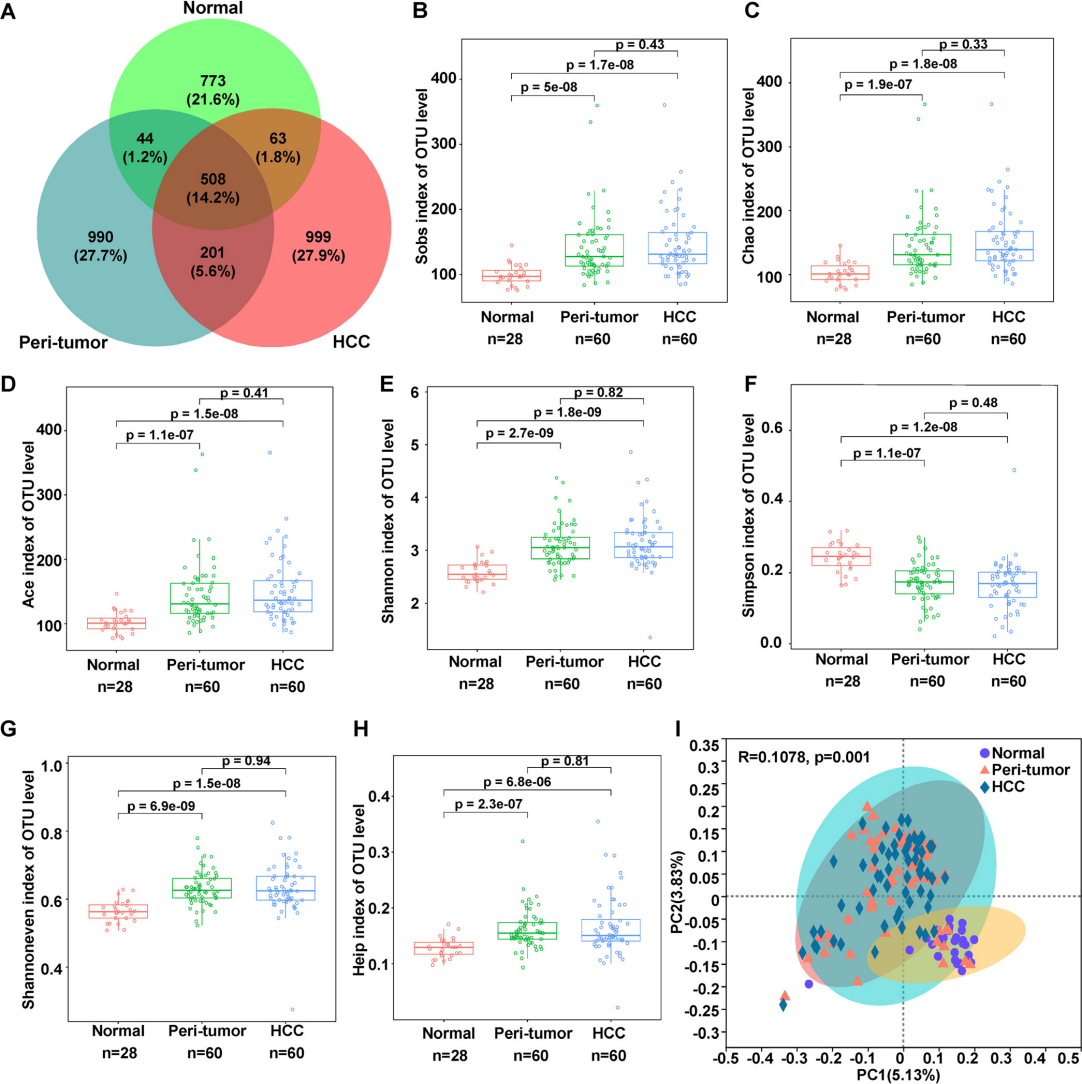
The α-diversities of NM, PtM, and HccM. A. The Venn diagram of the OTUs identified from NM, PtM, and HccM. B–H. The alpha diversity between three groups, including the richness indices Sobs (B), Chao (C), and Ace (D), diversity indices Shannon (E) and Simpson (F), and evenness indices Shannoneven (G) and Heip (H), were evaluated using two-sided Wilcoxon sum rank test. I. PCoA of binary Jaccard distance at the OTU level. The ellipses indicate the confidence interval (CI). The difference between the groups was tested using the Adonis algorithm with the number of replacements of 999.

The Venn diagram showed that 508 OTUs were shared among the three groups, while 709 OTUs were shared between the peritumoral microbiota (PtM) and the HCC microbiota (HccM) (Fig. 2A). PtM (Sobs_index/*P*_adj_ = 5e-8; Chao_index/*P*_adj_ = 1.9e-7; Ace_index/*P*_adj_ = 1.1e-7) and HccM (Sobs_index/*P*_adj_ = 1.7e-8; Chao_index, *P*_adj_ = 1.8e-8; Ace_index/*P*_adj_ = 1.5e-8) had significantly higher richness indices at the OTU or species level than the normal liver microbiota (NM) (Fig. 2B–D, Fig. S7A to C). Compared to NM, the diversity index Shannon was increased in PtM (OTU/*P*_adj_ = 2.7e-9) and HccM (OTU/*P*_adj_ = 1.8e-9), while Simpson was decreased in PtM (OTU/*P*_adj_ = 1.1e-7) and HccM (OTU/*P*_adj_ = 1.2e-8), which also indicated that PtM and HccM had a higher bacterial diversity (Fig. 2E and F, Fig. S7D and E). Furthermore, the evenness index of PtM (Shannoneven/*P*_adj_ = 6.9e-9; Heip/*P*_adj_ = 2.3e-7) and HccM (Shannoneven*/P*_adj_ = 1.5e-7; Heip/*P*_adj_ = 6.8e-6) were significantly higher than those of NM (Fig. 2G and H, Fig. S7F and G). No significant differences in alpha diversity were observed between PtM and HccM.

Principal coordinate analysis (PCoA) of the binary_jaccard distance at the OTU or species level revealed that NM was significantly different from PtM and HccM (OTU, *R* = 0.1078/Adonis *P* = 0.001) (Fig. 2I, Fig. S7H). Especially, eight peritumor samples were clustered with the normal samples, indicating their similarities in microbial features. Interestingly, the samples clustered closer together had lower Shannon diversity (especially the normal sample), whereas the more widely dispersed samples had higher Shannon diversity (Fig. S7I). Partial Least Squares Discriminant Analysis (PLS-DA) represents a discriminant analysis method for revealing specific bacterial taxa that contribute to major differences between different microhabitats. PLS-DA analysis performed at the phylum level revealed a similar bacterial profile separating the sample groups. Component one, which characterized the normal subjects with bacterial taxa *Acidobacteriota*, *Patescibacteria*, and others, accounted for 12.9% of the variance, while component three, which characterized the HCC subjects with *Proteobacteria*, *Patescibacteria*, and other taxa, accounted for 6.4% of the variance (Fig. S7J).

### The bacterial profile of HCC is significantly different from that of the normal liver.

**FIG 3:**
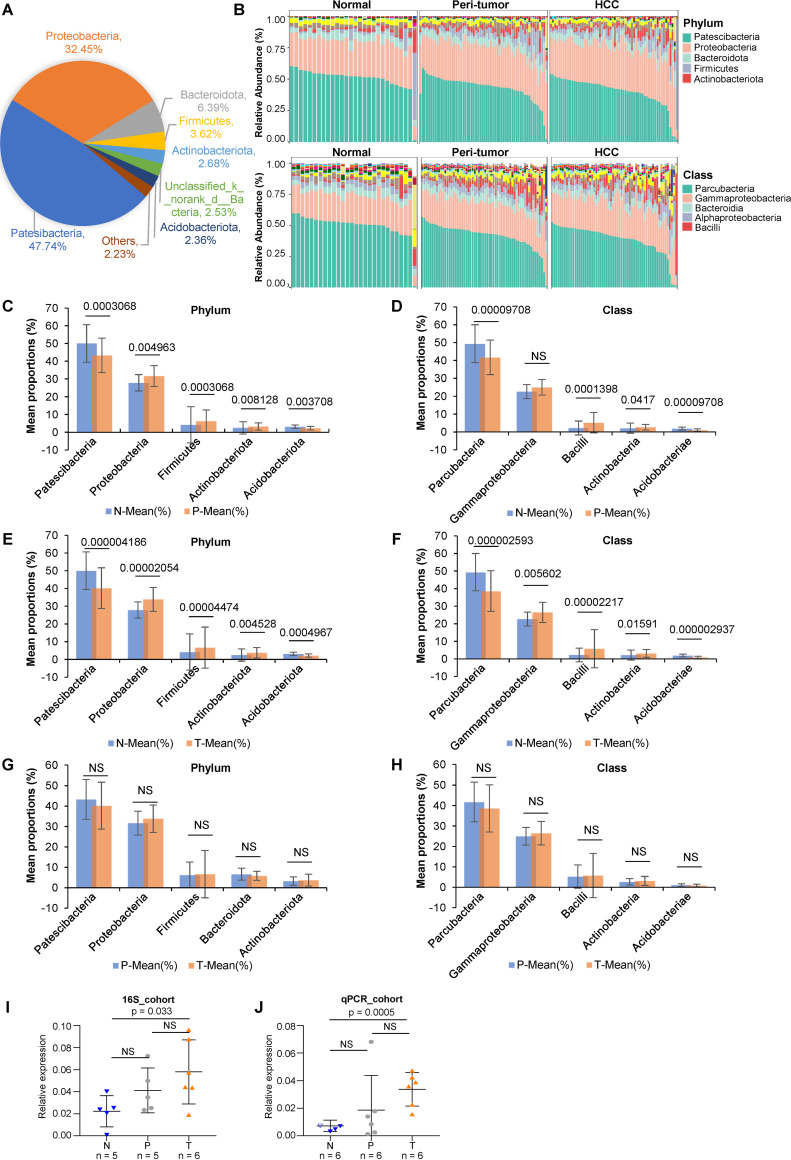
The profile of intratumoral microbes among normal, peritumor, and HCC tissues. A. The pie plot shows the proportion of each phylum in all liver microbiota based on OTU. B. The bar plot of the taxonomic composition at the phylum and class levels of NM, PtM, and HccM. C and D. Taxa abundance at the phylum or class level with significant difference between normal and peritumor microbiota. The significance was calculated using the two-tailed Wilcoxon sum rank test with continuity correction. The *P*-value was adjusted using the FDR method with 95% CI calculated using the bootstrap method. The adjusted *P*-values are shown on the top of the bars. E and F. Taxa abundance at the phylum or class level with significant difference between normal and HCC microbiota. G and H. Taxa abundance at the phylum or class level with significant difference between PtM and HccM. I and J. The abundances of *Gammaproteobacteria* were validated using qPCR analysis of the subjects randomly selected from the 16S_rRNA cohort (I) and an independent cohort (qPCR_cohort) (J) containing 6 peritumor (P) and 6 tumor (T) samples. The normal (N) samples measured were randomly selected from the 16S_rRNA cohort. The significance was calculated using unpaired and two-sided Student's *t* test. NS, nonsignificant.

The comparison of taxonomic abundance among NM, PtM, and HccM was listed in Table S2C to E. The phyla *Patesibacteria, Proteobacteria, Bacteroidota, Firmicutes,* and *Actinobacteriota* accounted for 92.88% of all taxa (Fig. 3A and B). At the class level, the top five classes were *Parcubacteria, Gammaproteobacteria, Bacteroidia, Alphaproteobacteria,* and *Bacilli*, from highest to lowest (Fig. 3B). The composition and abundance of bacteria at other levels were shown in Fig. S8. At the phylum level, PtM was enriched in *Proteobacteria* (*P*_adj_ = 0.0050; Wilcoxon Sum Rank test [similarly hereinafter]), *Firmicutes* (*P*_adj_ = 0.0003), and *Actinobacteriota* (*P*_adj_ = 0.0081), compared to NM, which showed higher levels of *Patescibacteria* (*P*_adj_ = 0.0003) and *Acidobacteriota* (*P*_adj_ = 0.0037) (Fig. 3C, Table S2C). At the class level, PtM was prevalent with *Bacilli* (*P*_adj_ = 0.0001) and *Actinobacteria* (*P*_adj_ = 0.0417), compared to NM, which contained significantly higher *Parcubacteria* (*P*_adj_ = 0.0001) and *Acidobacteriae* (*P*_adj_ = 0.0001) (Fig. 3D).

Regarding HCC, at the phylum level, HccM showed a higher prevalence of *Proteobacteria* (*P*_adj_ = 0.0000), *Firmicutes* (*P*_adj_ = 0.0000), and *Actinobacteriota* (*P*_adj_ = 0.0045), and reduced prevalence of *Patescibacteria* (*P*_adj_=0.0000) and *Acidobacteriota* (*P*_adj_ = 0.0005) compared to NM (Fig. 3E). At the class level, HccM was enriched for *Gammaproteobacteria* (*P*_adj_ = 0.0056), *Bacilli* (*P*_adj_ = 0.0000), and *Actinobacteria* (*P*_adj_ = 0.0159) compared to NM, which contained significantly higher levels of *Parcubacteria* (*P*_adj_ = 0.0000) and *Acidobacteriae* (*P*_adj_ = 0.0000) (Fig. 3F, Table S2D).

The above observations suggested that HccM and PtM had a quite similar profile of enriched or reduced taxa compared to NM. The five differential taxa (phyla or classes) were not statistically different between HccM and PtM (Fig. 3G and H). However, as of note, *Gammaproteobacteria* was significantly enriched in HccM but not PtM compared to NM. To verify the results of 16S rRNA sequencing, we performed a qPCR analysis of *Gammaproteobacteria* using samples from the 16S_rRNA cohort and an independent cohort containing 12 samples (qPCR_cohort) (Table S1). Consistent with the results of 16S rRNA gene sequencing, the abundance of *Gammaproteobacteria* in cancerous tissues was significantly higher (Student’ s *t* test, *P* = 0.033 in 16S_rRNA cohort or *P* = 0.0005 in qPCR_cohort) than in the normal samples (Fig. 3I and J). The peritumor tissues showed a higher average abundance of *Gammaproteobacteria* than normal tissues, although no statistical significance was achieved. Normal and peritumor samples did not differ significantly in terms of *Gammaproteobacteria.*

The enrichment of taxa at other taxonomic levels in HccM or PtM compared to NM was also shown in Table S2E. Heatmap analysis revealed the relationship between identified genera and the abundance across different liver microbiota (Fig. S9).

### Specific bacteria taxa of HccM revealed by the linear discriminant analysis (LDA) effect size (LEfSe) analysis.

**FIG 4:**
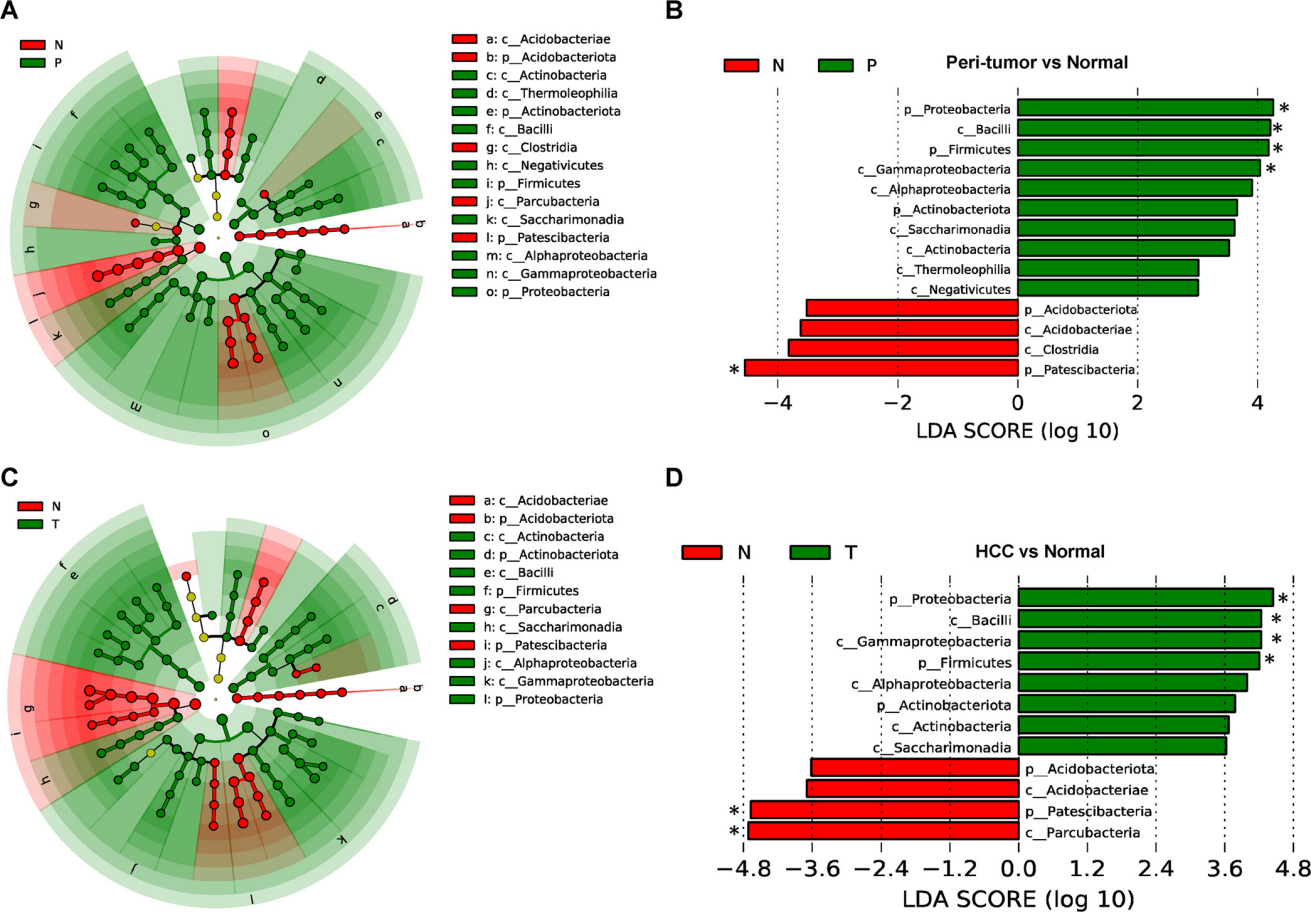
LEfSe analysis reveals the specific taxa of NM, PtM, and HccM. A. Cladogram showing the taxonomic tree of taxa with significantly differential abundance between normal (N) and peritumor (P) tissues. B. Histogram plot showing the linear discriminant analysis (LDA) scores (>3) of bacterial taxa with significantly differential abundance between normal and peritumor subjects. C. Cladogram showing the taxonomic tree of taxa with significantly differential abundance between normal (N) and HCC (T) tissues. D. Histogram plot showing the LDA scores (>3) of bacterial taxa with significant differential abundance between normal (N) and HCC subjects (T). The asterisks indicate the taxa with LDA scores > 4.

To further confirm the statistical differences and identify biological important taxa that distinguish different microhabitats, we performed LEfSe analysis with a logarithmic LDA score cutoff ≥3.0 (Table S3A). LEfSe results confirmed that the PtM of HCC patients had increased phyla *Proteobacteria*, *Firmicutes*, and *Actinobacteriota*, and classes *Bacilli*, *Gammaproteobacteria*, *Alphaproteobacteria*, S*accharimonadia*, *Actinobacteria*, *Thermoleophilia*, and *Negativicutes*, but decreased phyla *Protescibacteria* and *Acidobacteriota*, and classes *Acidobacteriae* and *Clostridia*, compared to NM controls (Fig. 4A and B). In HccM of HCC patients, the phyla *Proteobacteria*, *Firmicutes*, and *Actinobacteriota*, and classes *Bacilli*, *Gammaproteobacteria*, *Alphaproteobacteria*, *Saccharimonadia*, and *Actinobacteria*, were specifically enriched, whereas the phyla *Protescibacteria* and *Acidobacteriota* and classes *Parcubacteria* and *Acidobacteriae* were reduced, compared with NM controls (Fig. 4C and D).

In tissue culture, we have identified several viable species in peritumor or HCC specimens. Among these species, *Rothia* and *Kocuria* sp. belonged to the family *Micrococcaceae* (class *Actinobacteria*), which was found to be significantly enriched in both peritumor (LDA score = 3.10, Kruskal-Wallis test/*P*_adj_ = 0.0256) and HCC (LDA score = 3.35, *P*_adj_ = 0.0004) (Table S3B). Corynebacterium jeikeium, a member of the order *Corynebacteriales* (class *Actinobacteria*), was found to be enriched in both peritumor (LDA score = 3.27, *P*_adj_ = 0.0016) and HCC (LDA score = 3.38, *P*_adj_ = 0.0030). The viable species Cytobacillus horneckiae and S. aureus belonged to the class *Bacilli* (Table S2).

### Bacteria taxa showing differential abundance between HCCs with cirrhosis and HCCs without cirrhosis.

**FIG 5:**
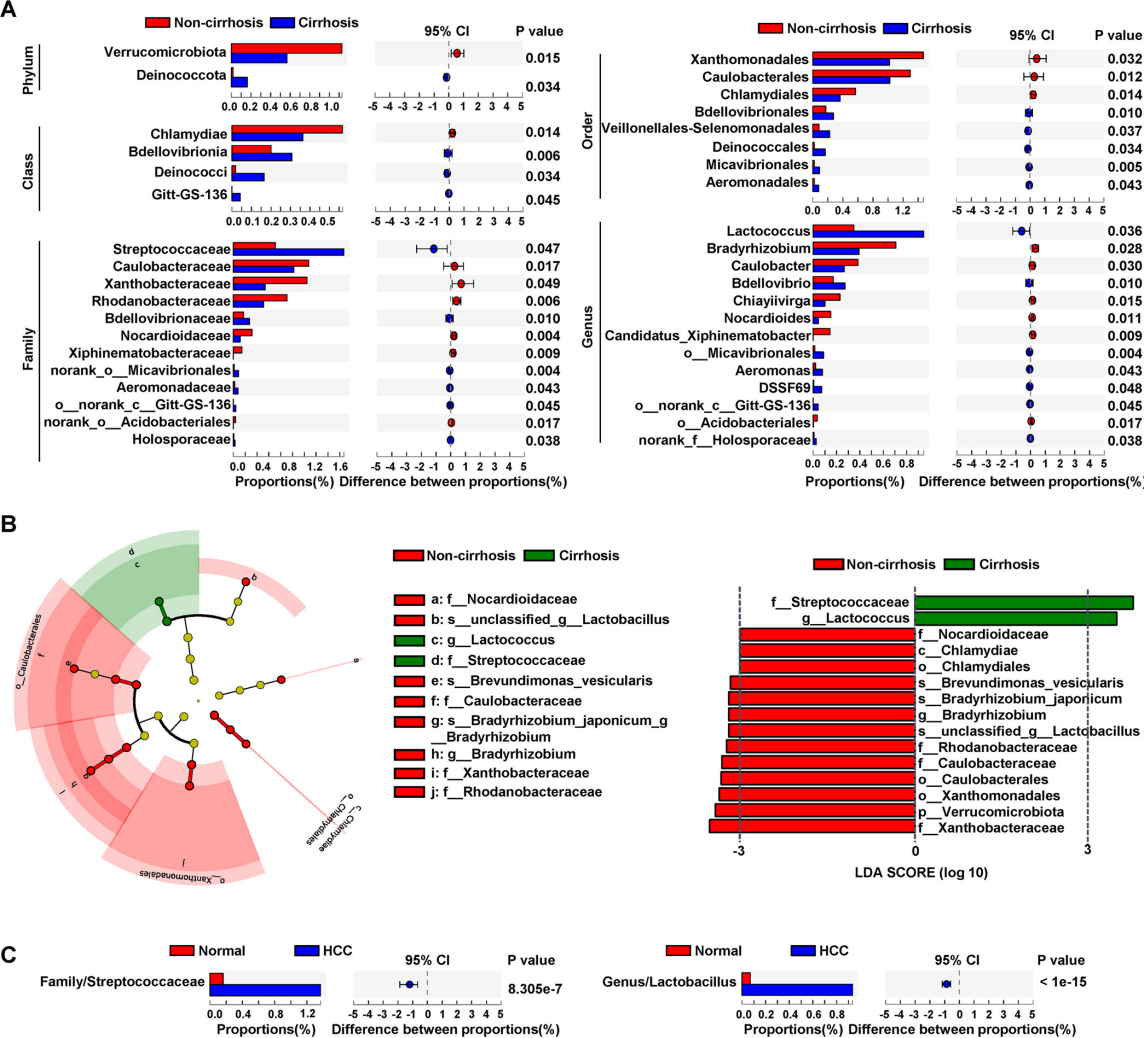
Bacterial taxa with differential abundance between HCCs with cirrhosis and HCCs without cirrhosis. A. The bacterial taxa showed a significant difference between cirrhosis (*n* = 34) and noncirrhosis (*n* = 26) HCC. The two-tailed *P*-value was calculated using Wilcoxon sum-rank test, and the *P*-value was adjusted using the FDR method. The 0.95% confidence intervals (CIs) were calculated using the bootstrap method. B. The cladogram and bar plot of LEfSe analysis shows the major taxa with the most abundance and a significant LDA score >3 in noncirrhosis and cirrhosis HCC. C. Abundance of *Streptococcaceae* and *Lactococcus* in normal liver and HCC tissues.

We analyzed the bacteria taxa that differed in abundance between cirrhotic (*n* = 28) and noncirrhotic (*n* = 34) HCC (Table S1A). Three HCCs were excluded from the analysis due to a low number of reads (<10,000). We found that the family *Streptococcaceae* (Wilcoxon test, adjusted *P* = 0.047) and the genus *Lactococcus* (*P* = 0.036) were significantly higher in cirrhotic HCCs than in noncirrhotic HCCs (Fig. 5A). On the other hand, the phylum *Verrucomicrobiota*, class *Chlamydiae*, order *Xanthomonadales* and *Caulobacterales*, family *Caulobacteraceae*, and genus *Bradyrhizobium* were significantly reduced in cirrhotic HCCs. We performed LEfSe analysis and revealed that *Streptococcaceae* and *Lactococcus* were the marker taxa of cirrhosis of HCC (Fig. 5B). *Streptococcaceae* and *Lactococcus* were significantly enriched in HCC tissues compared to normal liver tissues (Fig. 5C).

### Bacteria taxa enriched in HBV-negative HccM.

Although HBV represents the major viral oncogenic agent of HCC, there were HCCs of HBV-negative. Whether bacteria play a role in the pathological process of HBV-negative liver cancer remains to be studied. Wilcoxon sum-rank test revealed that the Staphylococcus branch and *Caulobacter* branch were selectively enriched in HBV-negative HCCs (*n* = 22) (Fig. S10A), which was further confirmed by LEfSe analysis (Fig. S10B). Interestingly, the *Caulobacter* branch was also identified as enriched taxa in noncirrhotic HccM, as shown above. On the other hand, a Streptococcus species was ranked as the top taxon in HBV-positive HCCs (Fig. S10B). The Streptococcus branch was also overrepresented in HccM with cirrhosis.

### Intratumoral microbial compositions correlate with the clinicopathological parameters of HCC patients.

Next, we asked whether the abundance of these key bacteria species was associated with the clinicopathological parameters of HCC patients. We found that the abundance of *Firmicutes* was correlated with cirrhosis inflammation grading (χ^2^ = 4.667, *P* = 0.0308) (Table S3C). The phylum *Actinobacteriota* showed a significant prevalence in female patients (χ^2^ = 6.6670, *P* = 0.0098) and was related to larger tumor volume (χ^2^ = 5.1923, *P* = 0.0027). At the class level, *Saccharimonadia* was related to cirrhosis inflammation grading (χ^2^ = 5.5100, *P* = 0.0189) and tumor volume (χ^2^ = 5.1923, *P = *0.0227). *Lactococcus* (genus) was significantly associated with cirrhosis status (χ^2^ = 6.7873, *P* = 0.0092) and HBcAb status (χ^2^ = 8.2162, *P* = 0.0042). These findings indicated that the intratumoral microbes were correlated with the clinicopathological features of HCC.

### Altered pathways in liver microbial communities.

**FIG 6:**
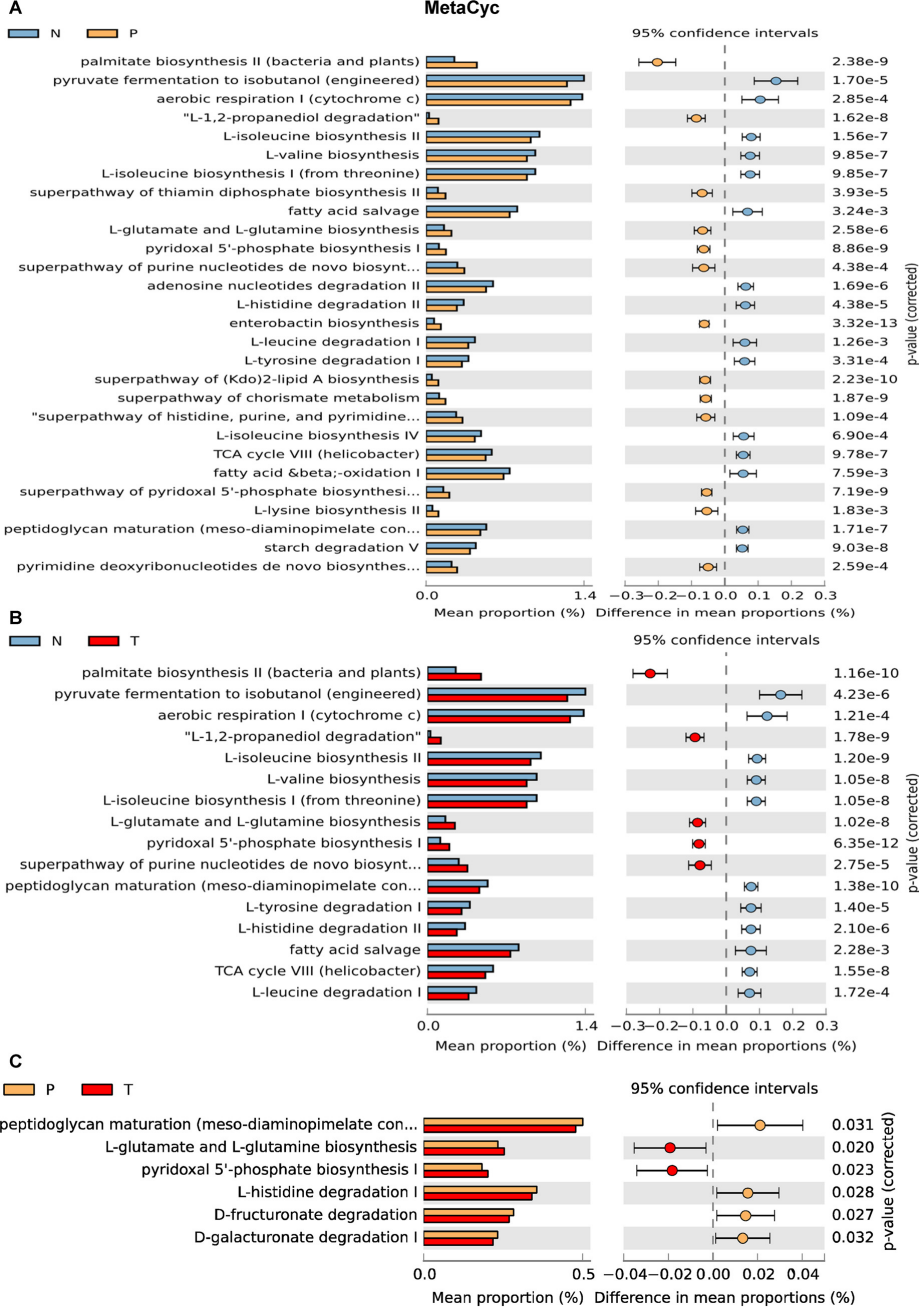
Inferred functions by PICRUSt across different liver microbiota. MetaCyc pathway analyses of different liver microbiota were performed using PICRUSt 2.0 (http://picrust.github.io/picrust/). The Kruskal-Wallis two-sided Welch’s t-test (threshold = 0.95) was used to test multiple comparisons of NM and PtM (A), NM and HccM (B), or PtM and HccM (C). N, normal liver; P, peritumor; T, tumor.

PiCRUSt2 was used to infer the function of the liver microbiota based on the OTU table. Analysis against MetaCyc metabolic pathway database revealed that 28 and 16 functional pathways were significantly changed between NM and PtM, and between NM and HccM, respectively (Fig. 6A and B). The palmitate biosynthesis pathway of bacteria was increased in both PtM (two-sided Welch’s t-test, *P = *2.38e-9) and HccM (two-sided Welch’s t-test, *P = *1.16e-10) compared to NM. In contrast, only six functional pathways were changed between PtM and HccM, indicating the similarity of these two microbiotas (Fig. 6C). The abundances of 11 clusters of orthologous groups (COG) functional categories in NM were significantly different from PtM (Fig. S11A). Furthermore, the 11 COGs showed differential abundance between NM and HccM (Fig. S11B). Compared with NM, three COGs were reduced in PtM and HccM, including energy production and conversion (Kruskal-Wallis test [similarly hereinafter], *P*_adj_ = 6.04e-5), amino acid transport and metabolism (*P*_adj_ = 1.46e-3), and signal transduction mechanism (*P*_adj_ = 3.17e-4), while four COGs were upregulated in PtM and HccM, including nucleotide transport and metabolism (*P*_adj_ = 1.72e-5), inorganic ion transport and metabolism, translation/ribosomal structure and biogenesis (*P*_adj_ = 1.69e-4), replication/recombination and repair (*P*_adj_ = 1.09e-5), and cell cycle control/cell division (*P*_adj_ = 7.31e-8) (Fig. S11C). Consistent with the alpha diversity analyses, PtM and HccM shared common functional features.

The results of the Kyoto Encyclopedia of Genes and Genome (KEGG) analysis at level 2 were the same as the COG analysis (Fig. S12A). Analysis of KEGG pathways at level three showed more metabolic changes between NM and PtM/HccM microbiota, including the upregulation of biosynthesis of amino acids in PtM/HccM (Fig. S12B). Taken together, the functional changes of different liver microbiota may play an important role in the progression of liver cancer.

### Identification of liver microbial signature for HCC prediction.

**FIG 7:**
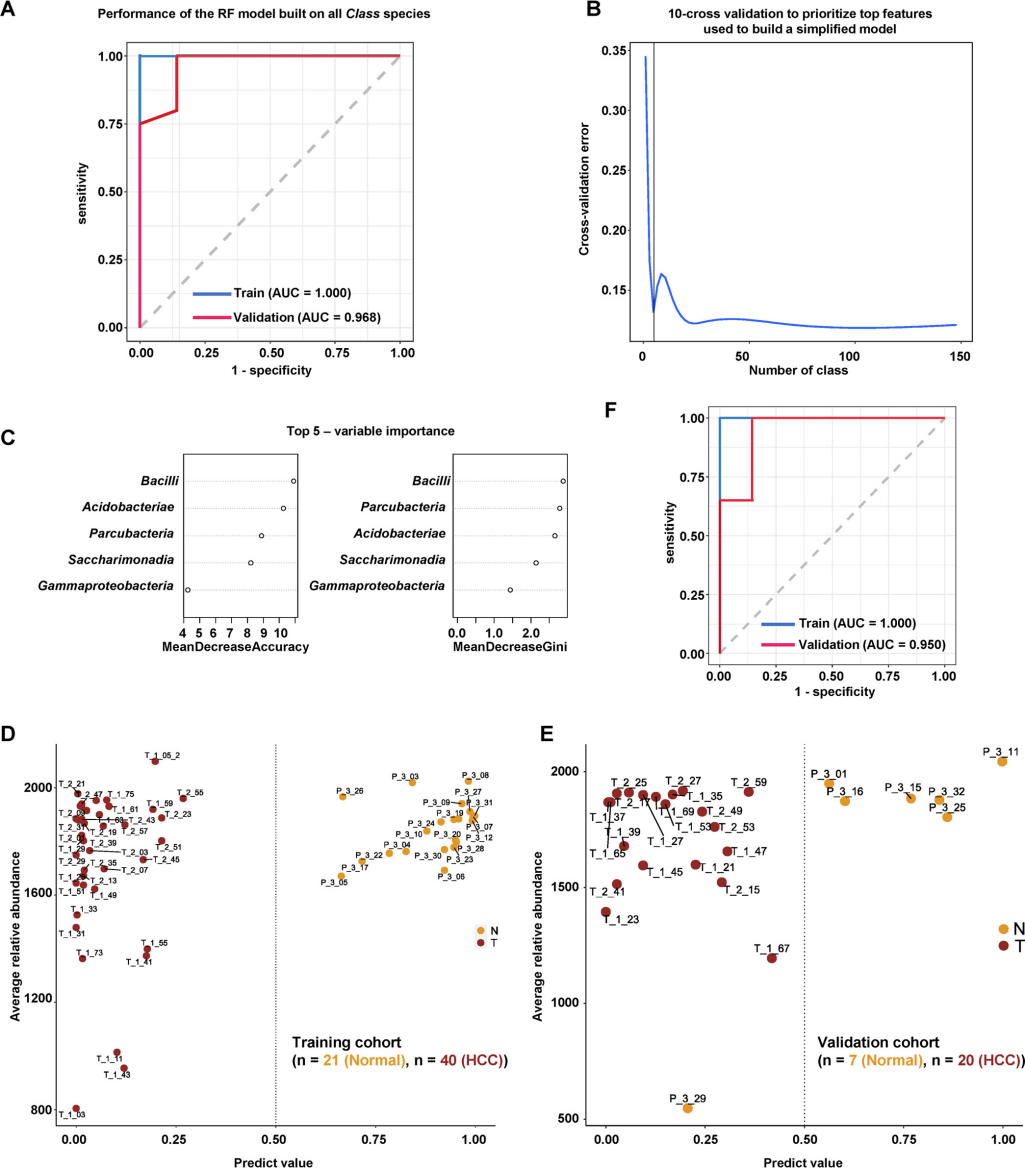
Microbial class-based diagnostic biomarker for HCC. A. Receiver operating characteristic (ROC) curves of the predictive performance of the class-based predictors for the RF model built on all class species. The blue and red curves indicated the performance of the model in the training cohort (HCC patient = 40, healthy = 20) and validation cohort (HCC patient = 20, healthy = 7), respectively. B. Cross-validation error curve shows the 10-fold cross validation method to prioritize top features used to build a simplified model. C. The top five class species prioritized by the 10-fold cross validation method. D. Performance of the simplified model using the top five class species in the training cohort containing 40 HCC patients and 20 healthy individuals. E. Performance of the simplified model using the top five class species in the validation cohort containing 20 HCC patients and 7 healthy individuals. F. ROC curves of predictive performance for the class-based predictors of the simplified RF model built on the top five class species. The blue and red curves indicate the performance of the model in the training cohort (HCC patient = 40, healthy = 20) and validation cohort (HCC patient = 20, healthy = 7), respectively.

The intratumoral microbial signatures may be useful for developing potential diagnostic or prognostic biomarkers for cancer. To build a clinical index for the diagnosis of HCC, we used a training cohort containing 40 HCC and 21 normal liver subjects to build a random forest (RF) prediction model using all microbial features at the class level. This model performed well not only in the training cohort (area under the curve [AUC] = 1.000) but also in the validation cohort containing 20 HCC and 7 normal subjects (AUC = 0.968) (Fig. 7A). To build a simplified model with reduced microbial signatures, we used 10-fold cross validation to prioritize the top features and selected the top five class species, including *Bacilli*, *Acidobacteriae*, *Parcubacteria*, *Saccharimonadia*, and *Gammaproteobacteria* (Fig. 7B and C). The reduced RF model separated HCC subjects from normal subjects in the training cohort (Fig. 7D), and its performance was verified in the validation cohort (Fig. 7E). The reduced model achieved comparable performance to the model using all class signatures (Fig. 7F). Since OTU-based predictors may be more accurate and reproducible (17), we analyzed the prediction power of the RF model using OTUs. First, we built an RF model using all 3,504 OTU features that achieved high prediction power (AUC = 1.000 in the training cohort and AUC = 0.939 in the validation cohort) (Fig. S13A). Using a 10-fold cross validation strategy, the top 50 OTU features were prioritized (Fig. S13B, C, Table S3D). The reduced RF model correctly predicted HCC subjects in both the training cohort and the validation cohort (Fig. S13D, E). The performance of the reduced model was comparable to that of the RF model using all OTU features (Fig. S13F).

## DISCUSSION

Gut microbiota is associated with the carcinogenesis and/or pathogenesis of human cancers, including liver cancer (5, 6). Furthermore, intratumoral bacteria have been found in a variety of human cancers, including pancreatic cancer, breast cancer, lung cancer, ovary cancer, melanoma, bone cancer, and brain cancers (13, 14). As one of the most important digestive organs, the liver has always been considered sterile. However, a recent study shows that the liver tissue of NAFLD contains a diverse repertoire of bacterial DNA (15). Here, we performed a FISH analysis using a bacterial 16S rRNA probe and revealed that liver RBCs were strongly stained in the peritumoral and tumor tissues. Blood is the carrier of the microbiome, which was confirmed using qPCR array analysis of the plasma from patients with cirrhosis (18). Mitchell et al. (19) argued that the pleomorphic structures in the blood are RBC-derived microparticles, not bacteria. However, conclusive evidence for the presence of bacteria in blood was provided by Paisse et al. (20) who prepared different blood fractions and analyzed bacterial 16S rRNA gene by real-time qPCR and MiSeq sequencing in a rigorous experimental setup. They revealed that RBCs contain more bacteria DNA than total plasma. Further evidence was provided by Damgaard et al. (21) who identified viable bacteria on plates inoculated with RBCs isolated from healthy individuals under anaerobic and aerobic culture conditions. Bacteria growth was observed in 21 of 60 RBC fractions in their cultures, and the colonies were identified using PCR and 16S rRNA gene sequencing. Viable bacteria, such as Streptococcus or Staphylococcus, have been found to inhabit the blood cells of healthy individuals (22, 23). Streptococcus pneumoniae can live intracellularly in RBCs to evade human innate immunity (24). These observations indicate that RBCs may represent important carriers of live bacteria into the liver, as we further illustrated in the current analysis.

Through the gut-liver microbiota axis, gut microbiota may modulate liver disease by transporting bacterial substances to the liver through the vascular and portal circulation systems (4, 5). Although another way bacteria entering the liver may be in the biliary tract where microbiota has been found (25–28), this pathway might not be as popular as the vascular pathway, because the fluorescence signal of the biliary tract was not visible in the FISH assay. Therefore, circulating cells like RBCs might be the potential carriers of bacterial substances into the liver. We found that the fluorescent signal of bacteria was enriched intracellularly in hepatocytes, especially in the cytosol rather than in the nucleus, which is consistent with the observations in other cancer types (13, 14). Since RBCs lack a nucleus, the FISH signal was spread throughout the RBCs. Nejman et al. (13) demonstrated by lipopolysaccharide (LPS) staining and transmission electron microscopy (TEM) that intratumoral bacteria lost their cell walls and localized in the cytosol and close to the nuclear membrane. These observations suggest that intracellular bacteria may exist in three forms: processed bacterial components like LPS, DNA, or lipoteichoic acid (LTA), bacteria without a cell wall, and bacteria with an intact cell wall, the latter of which may generate viable colonies in tissue cultures.

Fresh tissue culture provided prior evidence of the existence of live bacteria in human tumors (13). Furthermore, tissue culture coupled with d-alanine incorporation confirmed that breast cancer tumors harbor viable intracellular bacteria (13). For the first time, we found the presence of live bacteria in fresh peritumor and HCC tissues. Among the identified bacteria, S. aureus is a known pathogen of many severe diseases and its infection in the lung regulates metastasis of breast cancer cells (29), and it can invade and remain viable in white blood cells (22). S. aureus can also invade erythrocytes to evade host innate immunity (24). Rothia kristinae is an unusual pathogen that causes opportunistic infections in cancer patients (30, 31). C. horneckiae has been identified in cervical tissues from human papillomavirus-infected cervical cancers (32). All these viable species belong to the class *Bacilli* or *Actinobacteria*, and they were identified as taxonomic markers of HCC by LEfSe analysis.

We performed a comprehensive metataxonomic analysis of 155 human liver specimens. The alpha diversity of PtM and HccM was higher than that of NM. In PCoA, those samples with low taxonomic diversity (e.g., normal samples) tended to cluster closely, while those with high taxonomic diversity (e.g., HCC samples) tended to spread from each other, which suggests that increased diversity of microbiota may contribute to the heterogeneities of HCC. In contrast, the alpha diversity in PtM and HccM was not significantly different, suggesting that peritumor tissues have similar histological features to cancerous tissues, which may be due to their proximity to cancerous tissues. It has been reported that less diversity of microbial communities may be associated with worse disease, while higher diversity may indicate a healthy or better status. It was the case for the gastric mucosal microbiota, which showed a decreased diversity in cancer (33), while high diversity in pancreatic cancer was associated with better outcomes (14). However, this was not the case for breast cancer, where the abundance and richness were higher in the breast tumor samples than in normal adjacent (peritumoral) tissues and healthy breast tissues (13). Similarly, the diversity of gut microbes was also upregulated in HCC from cirrhosis to early cirrhosis (11). These observations suggest that disease progression may be related to the simplification or diversification of microbiota in the microhabitat.

We revealed that phyla *Proteobacteria, Bacteroidota, Firmicutes,* and *Actinobacteriota* were the most abundant taxa in liver microhabitats. *Patescibacteria* is a recently identified superphylum that represents a group of microbial communities living in freshwater environments (34). Interestingly, *Patescibacteria* also inhabits the human body, such as the lung (35), oral cavity (36), gut (37), and tumor tissues (38, 39). *Patescibacteria* was found to be the fourth dominant phylum of human adipose tissue (40). The relative abundance of gut *Patescibacteria* was 4-fold higher in pediatric multiple sclerosis compared to the monophasic acquired demyelinating syndrome (37). In disease models, gut *Patescibacteria* was found to be associated with antibiotic-induced diarrhea (41). In human cancer, *Patescibacteria* was found to be enriched in signet-ring cell carcinoma of gastric cancer (38). The abundance of oral *Patescibacteria* decreased significantly with the grade of glioma (42), while the phylum *Parcubacteria*, a lower taxon of *Patescibacteria*, was increased in rectal cancer compared to noncancer samples (39). These observations suggest that *Patescibacteria/Parcubacteria* may be associated with human diseases, including cancers, while its roles remain elusive.

Using statistical analysis and the LEfSe method, the Phyla *Proteobacteria*, *Firmicutes* and *Actinobacteriota*, and classes *Bacilli* and *Actinobacteria*, were consistently enriched in peritumor and HCC tissues, while *Gammaproteobacteria* was especially abundant in HCC tissues. Furthermore, by the Chi-square test, phyla *Firmicutes* and *Actinobacteriot*a, and class *Saccharimonadia* were found to be associated with the clinicopathological characteristics of HCC patients, including sex, cirrhosis grading (inflammation activity), or tumor volume, suggesting that these taxa might be linked to carcinogenesis or progression of HCC. Obesity increases the risk of HCC, and microbial derivatives promote HCC development (9). *Proteobacteria* was also upregulated in NAFLD (15), and its downstream branch, *Gammaproteobacteria*, was associated with histological severity in patients with morbidity NAFLD (15). The increased burden in HCC and the significant association with cirrhosis staging may indicate an important role for *Proteobacteria* and *Gammaproteobacteria* in the carcinogenesis or progression of HCC. Interestingly, a recent report has also found that *Proteobacteria* is one of the dominant phyla in liver cancer (43). Gut *Actinobacteriota* (phylum) was significantly increased in early HCC compared to liver cirrhosis (11). Given their correlation with tumor volume, both *Actinobacteriota* (phylum) and *Saccharimonadia* (class) may contribute to the progression of HCC.

We found that family *Streptococcaceae* and genus *Lactococcus* were significantly higher in HCCs with cirrhosis compared to HCCs without cirrhosis. Both taxa were also significantly increased in HCC tissues compared to normal liver tissues, suggesting these taxa may be associated with fibrosis and carcinogenesis of HCC. Interestingly, it is often found that gut *Streptococcaceae* is prevalent in patients with cirrhosis (44–46). Furthermore, Streptococcus gallolyticus is significantly associated with the occurrence of HCC (47). Streptococcus intermedius-derived histone-like DNA-binding protein was found to be involved in the pathogenesis of primary biliary cirrhosis (48). The abundance of *Lactococcus* was increased in gastric cancer (49). Ma et al. (50) showed that both Streptococcus and *Lactococcus* were significantly increased in prostatic fluid samples of prostate cancer patients compared to nonprostate cancer patients. Gao et al. (51) reported that the abundance of gut *Lactococcus* was relatively higher in colorectal cancer tissues compared to adjacent noncancerous tissues.

Chronic infection of HBV has been considered the dominant risk factor for HCC carcinogenesis. However, at least 20% of HCC does not attributable to HBV or HCV infection (52). An attractive question is, in the absence of a clear background of HBV or HCV infection, what are the potential bacterial agents that may contribute to the pathological process of HCC. We revealed that the Staphylococcus and *Caulobacter* branches were selectively enriched in HBV-negative HCCs. Interestingly, a recent nation-wide population-based study have demonstrated that the infection of S. aureus, an important opportunistic human pathogen, increases the risk of not only liver cancer but also other types of cancer (53). Noguchi et al. (54) revealed that a species of Staphylococcus might be associated with colon carcinoma. Our findings suggest that exposure to Staphylococcus infections, in addition to HBV, may increase the risk of liver cancer.

We analyzed the functional changes inferred from different liver microbiota. There were 11 COGs changed between NM, PtM, and HccM, which may indicate that the host nutrition and immune microhabitat may reshape microbial function, metabolism, and composition during colonization. The functional profiles of PtM and HccM were similar, which is consistent with the alpha diversity analyses, indicating that similar microhabitats determine similar microbial communities. Palmitate biosynthetic pathways were increased in PtM and HccM compared to NM. The ingestion of palmitate can activate the NF-κB pathway in hepatocytes (55) and promotes the development of HCC by increasing reactive oxygen species and subsequent glucose uptake (56). It should be noted that the functional analysis was based on indirect inference from 16S rRNA gene sequencing of the microbiome. Further analysis, such as metaproteomics, may provide insight into the function of the microbiota of the liver microenvironment since proteins are considered the final players of physiological functions.

Intratumoral microbiota signature may represent a novel and rich resource for developing potential biomarkers for cancer diagnosis. We built a machine learning model based on the class or OTU signature of HCC microbiota. The model displayed superior performance and accuracy in both the training and validation cohorts, suggesting that the intratumoral microbiota signature may have potential use in clinical settings. However, the number of subjects enrolled in the current study was limited, and the RF model should be evaluated using more samples in the future. The specific signature of intratumoral microbiota may also be useful in tumor staging and prognosis prediction.

There are some limitations in the current study. First, for ethical reasons, it is impossible to obtain many normal liver tissues from healthy volunteers. The normal liver tissues we used were derived from the normal part of the diseased liver. The normal liver tissues were confirmed by the treating surgeons to be free of disease. Furthermore, our results show that the microbiome of normal tissues differs significantly from that of paraneoplastic and HCCs. However, we cannot completely exclude the possibility that these normal tissues are affected by diseased liver tissues. Second, the paracancerous tissues of HCC were resected 2 cm away from cancer tissue. The distance may be too close and such paracancerous liver tissues were usually fibrotic or cirrhotic in nature. The similarity between PtM and HccM reduces the chance of finding increased oncogenic risk by comparing the two microbiotas. We used two independent cohorts to validate the sequencing results, while the heterogeneity of cancers may affect the congruence between different cohorts, which was especially the case for tissue cultures.

### Conclusion.

In summary, our analysis provides novel insights into the microbial characteristics of normal liver and HCC and reveals the association of specific taxa with the clinical features of HCC. We also develop a high-performance machine learning prediction model for accurate diagnosis of HCC.

## MATERIALS AND METHODS

### Clinical specimens and ethics.

Three cohorts were used in this study. The 16S_rRNA cohort used for 16S rRNA sequencing contained168 clinical tissue specimens from 100 individuals, including 68 paired primary HCC and peritumor HCC tissues (2 cm away from cancerous tissue), 3 additional peritumor tissues, and 29 cases of normal liver tissue. Of the HCC cases, 49 were HBV positive, while 21 HCCs were HBV negative, based on an immunoassay of surface antigen of HBV (HBsAg). These HCC patients were diagnosed according to multiple diagnostic results, including computerized tomography, B-mode ultrasonic diagnosis, magnetic resonance imaging, histological examination during surgery, and serological diagnosis like increased levels of serum AFP. Of the 71 HCC subjects, 40 cases of HCC were developed from liver cirrhosis (39 were S4 and one was cirrhosis, but GS was missing), and the others had no cirrhosis. The inclusion criteria were: (i) to ensure the yield of 16S rRNA sequencing, we collected fresh tissues isolated during the recent surgery (June 2019 to December 2019); (ii) Chinese patients; (iii) patients were diagnosed as HCC for the first time; and 4) patients were at least 18 years old. The exclusion criteria were: (i) HCC diagnosed 1 year before the time of the project; (ii) HCC patients with cancers diagnosed at other body sites. The clinical parameters of these patients were measured and recorded, including sex, age, grade (II, III, II–III), tumor count, tumor size of each tumor, inflammation grading (G1, G2, G3), cirrhosis fibrosis staging (S1, S2, S3, S4, S4e), tumor capsule (capsule present or not), nodule type (single nodule or multiple nodules), microvascular invasion (m0, m1, or m2), immunostaining for HBsAg antibody, immunostaining for HbcAb antibody, immunostaining for HCV antibody, and AFP concentration. The 29 normal liver tissues were isolated during surgery, including 18 liver metastases from colon adenocarcinoma, 4 angiomyolipomas, 2 focal nodular hyperplasias of the liver, one fibrous tissue hyperplasia, one liver metastasis from renal clear cell carcinoma, one liver metastasis from breast cancer, one cavernous hemangioma and one intraductal papillary neoplasm (Table S1). The normal liver tissues were resected at least 5 cm away from the tumorous tissue. The normal liver tissues were confirmed by clinical experts for their normal histological states. Due to ethical reasons, normal liver tissues cannot be obtained from healthy volunteers without any liver disease. In a previous study of single-cell sequencing of HCC, Aizarani et al. (57) also used nondiseased liver tissues as normal controls that had been resected from colorectal cancer metastases or cholangiocarcinoma.

An independent cohort of 12 tissue samples (qPCR_cohort) was used in quantitative PCR validation. The qPCR_cohort contained 6 HCC cancerous and 6 pericarcinoma specimens, isolated from 7 male and 3 female patients during clinical surgery (Table S1). The culturing cohort contained 12 paired pericarcinoma and HCC tissues from 11 males and one female patient (Table S1). One pair of tissues, 9P and 9T, was also used for H&E staining, Gram staining, and FISH analyses.

The third cohort (culturing cohort) contained12 paired pericarcinoma and cancerous tissue (HCC) samples that were used in tissue culture.

These specimens were collected at the Zhongshan Hospital, Fudan University, Shanghai, China. Written informed consent was obtained from all patients. The study was approved by the Research Ethics Committee of Zhongshan Hospital, Fudan University, Shanghai, China, and followed strictly the principles of the Declaration of Helsinki during the project.

### Tissue DNA extraction.

DNA of all tissues was extracted using the same protocol. Tissues (30 ~50 mg) were ground in disposable grinding tubes (2 mL in volume) with a steel ball (diameter 0.6 cm) in each tube. The grinding tube was prechilled with liquid nitrogen, and the tissues were ground for 10 s at a frequency of 50 Hz using a tissue grinder (WB2017075, Shanghai WonBio Biotechnology Co., Ltd.). DNA extraction was performed with the FastDNA spin kit for soil (MP Biomedicals, USA) according to the manufacturer’s instructions. DNA concentration and purity were measured using a NanoDrop 2000 UV-Vis spectrophotometer (Thermo Scientific, Wilmington, USA) according to the manufacturer’s instructions. The OD260/OD280 ratios of all DNA samples were greater than 1.9, indicating the good quality of the DNA samples. The average DNA concentration of all samples was 160.39 ± 84.87 ng/μL. The quality of the DNA samples was further evaluated using 1% agarose gel electrophoresis.

### 16S rRNA gene sequencing of negative controls.

To evaluate and exclude potential contaminant results, we sequenced negative-control samples collected during the DNA extraction and PCR amplification steps. The DNA extraction controls were extractions with blank extraction reagents without adding tissue samples. The extracted products were determined using agarose electrophoresis and the DNA concentrations of the DNA extraction controls were measured in the same way as the actual samples. A total 2 μL of DNA was used in the PCR experiments. The concentration of the PCR products was also measured.

The PCR negative controls were PCR experiments performed using distilled water as a template. The PCR products were checked using agarose electrophoresis and the DNA concentrations were determined. The extraction and PCR controls were subjected to MiSeq sequencing along with other samples.

We sequenced 6 extraction controls and 8 PCR controls, with one PCR control failing to yield sequencing results. The 16S rRNA gene sequencing results of the liver tissues were filtered by the R program decontam against the negative sequencing data using a frequency-based contaminant identification strategy (16). This strategy assumes that sequences from contaminating taxa have a higher prevalence in negative-control samples than in actual samples. After this step, s__Ralstonia_pickettii and s__unclassified_g__Sphingomonas were further removed from the actual sample results, since both taxa have more than 1,000 reads in the negative-control sequencing results.

### 16S rRNA gene PCR amplification.

The hypervariable V3-V4 region of bacterial 16S rRNA gene was amplified using primer pairs 341F (5′-CCTACGGGNGGCWGCAG-3′) and 805R (5′-GACTACHVGGGTATCTAATCC-3′) using TransStart Fastpfu DNA polymerase (TransGen AP221-02, TransGen Biotech Co.) in a GeneAmp PCR System 9700 Thermal Cycler (Applied Biosystems Inc., CA, USA). To ensure the accuracy and reliability of subsequent data analysis, we used low cycle number amplification whenever possible and used the same number of cycles for each sample. The reaction system consisted of 4 μL 5× FastPfu buffer, 2 μL dNTPs (2.5 mM), 0.8 μL forward primer (5 μM), 0.8 μL reverse primer (5 μM), 0.4 μL FastPfu polymerase, 0.2 μL BSA, and 10 ng template DNA. The volume was adjusted with water to 20 μL. The first-round PCR consisted of the following steps: 95°C for 3 min; 23 cycles (95°C for 30 s, 55°C for 30 s, and 72°C for 45 s); 72°C for 10 min; 10°C until halted by the user. The second-round PCR was performed to add index using the procedure: 95°C for 3 min; 8 cycles (95°C for 30 s, 55°C for 30 s, and 72°C for 45 s); 72°C for 10 min; 10°C, until halted by the user.

The amplification parameters were optimized in preliminary experiments. PCR products were analyzed using 2% agarose gel electrophoresis. PCR products were scored as A (strong), B (moderate), and C (weak or invisible) based on their size and concentration. Samples A and B were considered qualified samples and subjected to sequencing. For samples with a score of C, DNA extraction and PCR amplification were repeated using residual tissues of the same specimen. If PCR fails again, the sample was excluded from further analysis.

For subsequent sequencing, three PCR experiments were performed in parallel for each sample. PCR products of each sample were combined and subjected to 2% agarose gel electrophoresis. PCR fragments were recovered from the gels using the AxyPrep DNA gel extraction kit (AXYGEN Biosciences, Union City, CA, USA) according to the manufacturer’s instructions. DNA was eluted using Tris-HCl buffer, and its recovery rate and quality were evaluated by 2% agarose gel electrophoresis. DNA concentration was measured using the QuantiFluor-ST handheld fluorometer (Promega Corporation, Madison, WI, USA).

### 16S rRNA gene sequencing of normal liver, peritumor, and HCC samples.

To build a Miseq library for sequencing, Illumina adaptors were added to the termini of the target DNA by PCR using the TruSeq DNA sample prep kit according to the manufacturer’s instructions. PCR products were recovered from agarose gels using the AxyPrep DNA gel extraction kit. DNA was eluted using Tris-HCl buffer and the recovery rate and quality were evaluated using 2% agarose gel electrophoresis. Double-strand DNA was denatured using NaOH to generate single-strand DNA. Purified amplicons were pooled in equimolar and sequenced in paired-end mode on an Illumina MiSeq PE300 platform/NovaSeq PE250 platform (Illumina, San Diego, USA) according to the standard protocols by Majorbio Bio-Pharm Technology Co. Ltd. (Shanghai, China).

### Sequencing data processing and OTU clustering.

The raw 16S rRNA gene sequencing reads were demultiplexed, quality-filtered using Fastp version 0.20.0 (58) (https://github.com/OpenGene/fastp), and merged using FLASH version 1.2.11 (59) (https://ccb.jhu.edu/software/FLASH/index.shtml). First, the low-quality tails of each sequence with an average quality score of <20 over a 50-bp sliding window were truncated. After tail truncation, reads containing ambiguous characters or <50 bp in length were discarded. Cleaned reads with at least 10 bp of overlapping sequences were then assembled. The maximum mismatch ratio for the overlap region is 0.2. Singleton reads that could not be assembled were discarded. The assembled sequences were distinguished based on barcode sequences and primers. No mismatches of barcodes were allowed. Two mismatched nucleotides were allowed for primers. Finally, the sequence direction was adjusted.

OTUs with 97% similarity were clustered using UPARSE version 7.1 (60) (http://www.drive5.com/uparse/), and chimeric sequences were identified and removed. The OTU table was used in all downstream analyses. The taxonomy of each OTU representative sequence was analyzed by RDP Classifier version 2.11 (https://sourceforge.net/projects/rdp-classifier/) against the 16S rRNA database Silva 138 (https://www.arb-silva.de/) using a confidence threshold of 0.7 (61). The taxonomic composition was analyzed at the level of domain, kingdom, p, class, order, family, genus, and species.

### Analyzing 16S rRNA sequencing data.

Data were analyzed on the online Majorbio Cloud platform (www.majorbio.com), which has integrated all downstream tools for microbiota analysis. First, we removed the order level chloroplast sequences (o_Chloroplast) from the original OTU table. We flattened the OTU table according to the minimum number of sample sequences (11,807 reads). Flattening is the random selection of sequences of all samples as a uniform amount of data according to a certain number or the minimum number of sequences in each sample. The rank-abundance curve indicating the diversity and evenness of microbiota was generated using R scripts. Alpha diversity was calculated using Mothur v1.30.2 (https://www.mothur.org/wiki/Download_mothur). The alpha diversity indices include community richness (Sobs, Chao, Ace), community evenness (Simpsoneven, Heip), and community diversity (Shannon, Simpson). The significance of the alpha diversity difference between different clinic groups was calculated using the Wilcoxon rank-sum test. The rarefaction curve is used to determine whether the amount of sequencing data are sufficient according to whether the curve is flat. Curves based on alpha diversity values were generated using R scripts.

Beta diversity was measured using the binary Jaccard distance calculated by Qiime 1.9.1 (http://qiime.org/install/index.html). The distances were visualized using PCoA. Differences between groups were tested using the Adonis algorithm with a replacement number of 999. PLS-DA was conducted using the mixOmics package of the R program to reveal the most predictive/discriminative taxa for classifying each group.

Differences in taxonomic abundance between ≥3 groups of microhabitats or clinical features were calculated using the Kruskal-Wallis H test and the *P*-value was adjusted using the false-discovery rate (FDR) method. The 0.95% confidence intervals were calculated using the Tukey-Kramer method. For taxonomic differences between the two groups, two-tailed *P*-values were calculated using Wilcoxon sum-rank test, and *P*-values were adjusted using the FDR method. The 0.95% confidence intervals were calculated using the bootstrap method.

LEfSe was performed on the Majorbio Cloud platform. *P*-values were calculated using the nonparametric factorial Kruskal-Wallis sum-rank test, and the threshold for the logarithmic LDA score of discriminative features was set to 3.0. The strategy for multiclass analysis was all-against-all. The result table was downloaded as a lefse_internal_res file, which was further edited and subjected for LEfSe visualization on the online Galaxy server (http://huttenhower.sph.harvard.edu/galaxy/). The histogram and cladogram figures were generated by the LEfSe visualization modules. The alpha values of the factorial Kruskal-Wallis test among classes and the pairwise Wilcoxon test between subclasses were set to 0.05.

COG, KEGG, MetaCyc pathway, and enzyme category function analyses were performed using PICRUSt 2.0 (http://picrust.github.io/picrust/). The significance of the pairwise comparison between groups was evaluated with the two-sided Welch’s t-test, and 95% confidence intervals for differences in mean proportion were calculated using the Welch’s inverted method. Comparisons of function categories between normal, peritumor, and HCC groups were evaluated with the Kruskal-Wallis test followed by the *post hoc* Tukey-Kramer test (threshold = 0.95) using STAMP 2.1.3 (https://beikolab.cs.dal.ca/software/STAMP).

### Data availability.

The data set supporting the conclusions of this article is available in the GenBank Sequence Read Archive under BioProject ID PRJNA714196.

Other methods were available in TEXTS1.

## References

[1] Kanwal F, Singal AG. 2019. Surveillance for hepatocellular carcinoma: current best practice and future direction. Gastroenterology 157:54–64. doi:10.1053/j.gastro.2019.02.049. 30986389 PMC6636644

[2] Bray F, Ferlay J, Soerjomataram I, Siegel RL, Torre LA, Jemal A. 2018. Global cancer statistics 2018: GLOBOCAN estimates of incidence and mortality worldwide for 36 cancers in 185 countries. CA Cancer J Clin 68:394–424. doi:10.3322/caac.21492. 30207593

[3] de Martel C, Georges D, Bray F, Ferlay J, Clifford GM. 2020. Global burden of cancer attributable to infections in 2018: a worldwide incidence analysis. Lancet Glob Health 8:e180–e190. doi:10.1016/S2214-109X(19)30488-7. 31862245

[4] Bawa M, Saraswat VA. 2013. Gut-liver axis: role of inflammasomes. J Clin Exp Hepatol 3:141–149. doi:10.1016/j.jceh.2013.03.225. 25755488 PMC4216435

[5] Ponziani FR, Bhoori S, Castelli C, Putignani L, Rivoltini L, Del Chierico F, Sanguinetti M, Morelli D, Paroni Sterbini F, Petito V, Reddel S, Calvani R, Camisaschi C, Picca A, Tuccitto A, Gasbarrini A, Pompili M, Mazzaferro V. 2019. Hepatocellular carcinoma is associated with gut microbiota profile and inflammation in nonalcoholic fatty liver disease. Hepatology 69:107–120. doi:10.1002/hep.30036. 29665135

[6] Yip LY, Aw CC, Lee SH, Hong YS, Ku HC, Xu WH, Chan JMX, Cheong EJY, Chng KR, Ng AHQ, Nagarajan N, Mahendran R, Lee YK, Browne ER, Chan ECY. 2018. The liver-gut microbiota axis modulates hepatotoxicity of tacrine in the rat. Hepatology 67:282–295. doi:10.1002/hep.29327. 28646502

[7] Dapito DH, Mencin A, Gwak GY, Pradere JP, Jang MK, Mederacke I, Caviglia JM, Khiabanian H, Adeyemi A, Bataller R, Lefkowitch JH, Bower M, Friedman R, Sartor RB, Rabadan R, Schwabe RF. 2012. Promotion of hepatocellular carcinoma by the intestinal microbiota and TLR4. Cancer Cell 21:504–516. doi:10.1016/j.ccr.2012.02.007. 22516259 PMC3332000

[8] Ma C, Han M, Heinrich B, Fu Q, Zhang Q, Sandhu M, Agdashian D, Terabe M, Berzofsky JA, Fako V, Ritz T, Longerich T, Theriot CM, McCulloch JA, Roy S, Yuan W, Thovarai V, Sen SK, Ruchirawat M, Korangy F, Wang XW, Trinchieri G, Greten TF. 2018. Gut microbiome-mediated bile acid metabolism regulates liver cancer via NKT cells. Science 360. doi:10.1126/science.aan5931. PMC6407885 29798856

[9] Loo TM, Kamachi F, Watanabe Y, Yoshimoto S, Kanda H, Arai Y, Nakajima-Takagi Y, Iwama A, Koga T, Sugimoto Y, Ozawa T, Nakamura M, Kumagai M, Watashi K, Taketo MM, Aoki T, Narumiya S, Oshima M, Arita M, Hara E, Ohtani N. 2017. Gut microbiota promotes obesity-associated liver cancer through PGE2-mediated suppression of antitumor immunity. Cancer Discov 7:522–538. doi:10.1158/2159-8290.CD-16-0932. 28202625

[10] Yoshimoto S, Loo TM, Atarashi K, Kanda H, Sato S, Oyadomari S, Iwakura Y, Oshima K, Morita H, Hattori M, Hattori M, Honda K, Ishikawa Y, Hara E, Ohtani N. 2013. Obesity-induced gut microbial metabolite promotes liver cancer through senescence secretome. Nature 499:97–101. doi:10.1038/nature12347. 23803760

[11] Ren Z, Li A, Jiang J, Zhou L, Yu Z, Lu H, Xie H, Chen X, Shao L, Zhang R, Xu S, Zhang H, Cui G, Chen X, Sun R, Wen H, Lerut JP, Kan Q, Li L, Zheng S. 2019. Gut microbiome analysis as a tool towards targeted non-invasive biomarkers for early hepatocellular carcinoma. Gut 68:1014–1023. doi:10.1136/gutjnl-2017-315084. 30045880 PMC6580753

[12] Qin N, Yang F, Li A, Prifti E, Chen Y, Shao L, Guo J, Le Chatelier E, Yao J, Wu L, Zhou J, Ni S, Liu L, Pons N, Batto JM, Kennedy SP, Leonard P, Yuan C, Ding W, Chen Y, Hu X, Zheng B, Qian G, Xu W, Ehrlich SD, Zheng S, Li L. 2014. Alterations of the human gut microbiome in liver cirrhosis. Nature 513:59–64. doi:10.1038/nature13568. 25079328

[13] Nejman D, Livyatan I, Fuks G, Gavert N, Zwang Y, Geller LT, Rotter-Maskowitz A, Weiser R, Mallel G, Gigi E, Meltser A, Douglas GM, Kamer I, Gopalakrishnan V, Dadosh T, Levin-Zaidman S, Avnet S, Atlan T, Cooper ZA, Arora R, Cogdill AP, Khan MAW, Ologun G, Bussi Y, Weinberger A, Lotan-Pompan M, Golani O, Perry G, Rokah M, Bahar-Shany K, Rozeman EA, Blank CU, Ronai A, Shaoul R, Amit A, Dorfman T, Kremer R, Cohen ZR, Harnof S, Siegal T, Yehuda-Shnaidman E, Gal-Yam EN, Shapira H, Baldini N, Langille MGI, Ben-Nun A, Kaufman B, Nissan A, Golan T, Dadiani M, et al. 2020. The human tumor microbiome is composed of tumor type-specific intracellular bacteria. Science 368:973–980. doi:10.1126/science.aay9189. 32467386 PMC7757858

[14] Riquelme E, Zhang Y, Zhang L, Montiel M, Zoltan M, Dong W, Quesada P, Sahin I, Chandra V, San Lucas A, Scheet P, Xu H, Hanash SM, Feng L, Burks JK, Do KA, Peterson CB, Nejman D, Tzeng CD, Kim MP, Sears CL, Ajami N, Petrosino J, Wood LD, Maitra A, Straussman R, Katz M, White JR, Jenq R, Wargo J, McAllister F. 2019. Tumor microbiome diversity and composition influence pancreatic cancer outcomes. Cell 178:795–806.e712. doi:10.1016/j.cell.2019.07.008. 31398337 PMC7288240

[15] Sookoian S, Salatino A, Castano GO, Landa MS, Fijalkowky C, Garaycoechea M, Pirola CJ. 2020. Intrahepatic bacterial metataxonomic signature in non-alcoholic fatty liver disease. Gut 69:1483–1491. doi:10.1136/gutjnl-2019-318811. 31900291

[16] Davis NM, Proctor DM, Holmes SP, Relman DA, Callahan BJ. 2018. Simple statistical identification and removal of contaminant sequences in marker-gene and metagenomics data. Microbiome 6:226. doi:10.1186/s40168-018-0605-2. 30558668 PMC6298009

[17] Zheng Y, Fang Z, Xue Y, Zhang J, Zhu J, Gao R, Yao S, Ye Y, Wang S, Lin C, Chen S, Huang H, Hu L, Jiang GN, Qin H, Zhang P, Chen J, Ji H. 2020. Specific gut microbiome signature predicts the early-stage lung cancer. Gut Microbes 11:1030–1042. doi:10.1080/19490976.2020.1737487. 32240032 PMC7524275

[18] Traykova D, Schneider B, Chojkier M, Buck M. 2017. Blood microbiome quantity and the hyperdynamic circulation in decompensated cirrhotic patients. PLoS One 12:e0169310. doi:10.1371/journal.pone.0169310. 28146577 PMC5287452

[19] Mitchell AJ, Gray WD, Schroeder M, Yi H, Taylor JV, Dillard RS, Ke Z, Wright ER, Stephens D, Roback JD, Searles CD. 2016. Pleomorphic structures in human blood are red blood cell-derived microparticles, not bacteria. PLoS One 11:e0163582. doi:10.1371/journal.pone.0163582. 27760197 PMC5070825

[20] Paisse S, Valle C, Servant F, Courtney M, Burcelin R, Amar J, Lelouvier B. 2016. Comprehensive description of blood microbiome from healthy donors assessed by 16S targeted metagenomic sequencing. Transfusion 56:1138–1147. doi:10.1111/trf.13477. 26865079

[21] Damgaard C, Magnussen K, Enevold C, Nilsson M, Tolker-Nielsen T, Holmstrup P, Nielsen CH. 2015. Viable bacteria associated with red blood cells and plasma in freshly drawn blood donations. PLoS One 10:e0120826. doi:10.1371/journal.pone.0120826. 25751254 PMC4353618

[22] Yamaguchi H, Yamada M, Uruma T, Kanamori M, Goto H, Yamamoto Y, Kamiya S. 2004. Prevalence of viable Chlamydia pneumoniae in peripheral blood mononuclear cells of healthy blood donors. Transfusion 44:1072–1078. doi:10.1111/j.1537-2995.2004.04005.x. 15225250

[23] Gresham HD, Lowrance JH, Caver TE, Wilson BS, Cheung AL, Lindberg FP. 2000. Survival of Staphylococcus aureus inside neutrophils contributes to infection. J Immunol 164:3713–3722. doi:10.4049/jimmunol.164.7.3713. 10725730

[24] Yamaguchi M, Terao Y, Mori-Yamaguchi Y, Domon H, Sakaue Y, Yagi T, Nishino K, Yamaguchi A, Nizet V, Kawabata S. 2013. Streptococcus pneumoniae invades erythrocytes and utilizes them to evade human innate immunity. PLoS One 8:e77282. doi:10.1371/journal.pone.0077282. 24194877 PMC3806730

[25] Wu T, Zhang Z, Liu B, Hou D, Liang Y, Zhang J, Shi P. 2013. Gut microbiota dysbiosis and bacterial community assembly associated with cholesterol gallstones in large-scale study. BMC Genomics 14:669. doi:10.1186/1471-2164-14-669. 24083370 PMC3851472

[26] Molinero N, Ruiz L, Milani C, Gutierrez-Diaz I, Sanchez B, Mangifesta M, Segura J, Cambero I, Campelo AB, Garcia-Bernardo CM, Cabrera A, Rodriguez JI, Gonzalez S, Rodriguez JM, Ventura M, Delgado S, Margolles A. 2019. The human gallbladder microbiome is related to the physiological state and the biliary metabolic profile. Microbiome 7:100. doi:10.1186/s40168-019-0712-8. 31272480 PMC6610825

[27] Shen H, Ye F, Xie L, Yang J, Li Z, Xu P, Meng F, Li L, Chen Y, Bo X, Ni M, Zhang X. 2015. Metagenomic sequencing of bile from gallstone patients to identify different microbial community patterns and novel biliary bacteria. Sci Rep 5:17450. doi:10.1038/srep17450. 26625708 PMC4667190

[28] Kose SH, Grice K, Orsi WD, Ballal M, Coolen MJL. 2018. Metagenomics of pigmented and cholesterol gallstones: the putative role of bacteria. Sci Rep 8:11218. doi:10.1038/s41598-018-29571-8. 30046045 PMC6060111

[29] Qi JL, He JR, Liu CB, Jin SM, Gao RY, Yang X, Bai HM, Ma YB. 2020. Pulmonary Staphylococcus aureus infection regulates breast cancer cell metastasis via neutrophil extracellular traps (NETs) formation. MedComm (2020) 1:188–201. doi:10.1002/mco2.22. 34766117 PMC8491238

[30] Chen HM, Chi H, Chiu NC, Huang FY. 2015. Kocuria kristinae: a true pathogen in pediatric patients. J Microbiol Immunol Infect 48:80–84. doi:10.1016/j.jmii.2013.07.001. 23968754

[31] Basaglia G, Carretto E, Barbarini D, Moras L, Scalone S, Marone P, De Paoli P. 2002. Catheter-related bacteremia due to Kocuria kristinae in a patient with ovarian cancer. J Clin Microbiol 40:311–313. doi:10.1128/JCM.40.1.311-313.2002. 11773142 PMC120093

[32] Kwasniewski W, Wolun-Cholewa M, Kotarski J, Warchol W, Kuzma D, Kwasniewska A, Gozdzicka-Jozefiak A. 2018. Microbiota dysbiosis is associated with HPV-induced cervical carcinogenesis. Oncol Lett 16:7035–7047. doi:10.3892/ol.2018.9509. 30546437 PMC6256731

[33] Liu X, Shao L, Liu X, Ji F, Mei Y, Cheng Y, Liu F, Yan C, Li L, Ling Z. 2019. Alterations of gastric mucosal microbiota across different stomach microhabitats in a cohort of 276 patients with gastric cancer. EBioMedicine 40:336–348. doi:10.1016/j.ebiom.2018.12.034. 30584008 PMC6412016

[34] Buck M, Garcia SL, Fernandez L, Martin G, Martinez-Rodriguez GA, Saarenheimo J, Zopfi J, Bertilsson S, Peura S. 2021. Comprehensive dataset of shotgun metagenomes from oxygen stratified freshwater lakes and ponds. Sci Data 8:131. doi:10.1038/s41597-021-00910-1. 33990618 PMC8121793

[35] de Dios Caballero J, Vida R, Cobo M, Maiz L, Suarez L, Galeano J, Baquero F, Canton R, Del Campo R. 2017. Individual patterns of complexity in cystic fibrosis lung microbiota, including predator bacteria, over a 1-year period. mBio 8. doi:10.1128/mBio.00959-17. PMC5615197 28951476

[36] Alqaderi H, Ramakodi MP, Nizam R, Jacob S, Devarajan S, Eaaswarkhanth M, Al-Mulla F. 2021. Salivary microbiome diversity in Kuwaiti adolescents with varied body mass index-A pilot study. Microorganisms 9:1222. doi:10.3390/microorganisms9061222. 34200004 PMC8228046

[37] Tremlett H, Zhu F, Arnold D, Bar-Or A, Bernstein CN, Bonner C, Forbes JD, Graham M, Hart J, Knox NC, Marrie RA, Mirza AI, O’Mahony J, Van Domselaar G, Yeh EA, Zhao Y, Banwell B, Waubant E, Us Network of Pediatric Ms Centers tCPDDN. 2021. The gut microbiota in pediatric multiple sclerosis and demyelinating syndromes. Ann Clin Transl Neurol 8:2252–2269. doi:10.1002/acn3.51476. 34889081 PMC8670321

[38] Ravegnini G, Fosso B, Saverio VD, Sammarini G, Zanotti F, Rossi G, Ricci M, D'Amico F, Valori G, Ioli A, Turroni S, Brigidi P, Hrelia P, Angelini S. 2020. Gastric adenocarcinomas and signet-ring cell carcinoma: unraveling gastric cancer complexity through microbiome analysis-deepening heterogeneity for a personalized therapy. Int J Mol Sci 21:9735. doi:10.3390/ijms21249735. 33419357 PMC7766162

[39] Thomas AM, Jesus EC, Lopes A, Aguiar S, Jr, Begnami MD, Rocha RM, Carpinetti PA, Camargo AA, Hoffmann C, Freitas HC, Silva IT, Nunes DN, Setubal JC, Dias-Neto E. 2016. Tissue-associated bacterial alterations in rectal carcinoma patients revealed by 16s rRNA community profiling. Front Cell Infect Microbiol 6:179. 28018861 10.3389/fcimb.2016.00179 PMC5145865

[40] Massier L, Chakaroun R, Tabei S, Crane A, Didt KD, Fallmann J, von Bergen M, Haange SB, Heyne H, Stumvoll M, Gericke M, Dietrich A, Bluher M, Musat N, Kovacs P. 2020. Adipose tissue derived bacteria are associated with inflammation in obesity and type 2 diabetes. Gut 69:1796–1806. doi:10.1136/gutjnl-2019-320118. 32317332

[41] Ding Z, Wang W, Zhang K, Ming F, Yangdai T, Xu T, Shi H, Bao Y, Yao H, Peng H, Han C, Jiang W, Liu J, Hou X, Lin R. 2021. Novel scheme for non-invasive gut bioinformation acquisition with a magnetically controlled sampling capsule endoscope. Gut 70:2297–2306. doi:10.1136/gutjnl-2020-322465. 33452177

[42] Wen Y, Feng L, Wang H, Zhou H, Li Q, Zhang W, Wang M, Li Y, Luan X, Jiang Z, Chen L, Zhou J. 2021. Association between oral microbiota and human brain glioma grade: a case-control study. Front Microbiol 12:746568. doi:10.3389/fmicb.2021.746568. 34733261 PMC8558631

[43] Komiyama S, Yamada T, Takemura N, Kokudo N, Hase K, Kawamura YI. 2021. Profiling of tumour-associated microbiota in human hepatocellular carcinoma. Sci Rep 11:10589. doi:10.1038/s41598-021-89963-1. 34012007 PMC8134445

[44] Chen Y, Yang F, Lu H, Wang B, Chen Y, Lei D, Wang Y, Zhu B, Li L. 2011. Characterization of fecal microbial communities in patients with liver cirrhosis. Hepatology 54:562–572. doi:10.1002/hep.24423. 21574172

[45] Bajaj JS, Cox IJ, Betrapally NS, Heuman DM, Schubert ML, Ratneswaran M, Hylemon PB, White MB, Daita K, Noble NA, Sikaroodi M, Williams R, Crossey MM, Taylor-Robinson SD, Gillevet PM. 2014. Systems biology analysis of omeprazole therapy in cirrhosis demonstrates significant shifts in gut microbiota composition and function. Am J Physiol Gastrointest Liver Physiol 307:G951–G957. doi:10.1152/ajpgi.00268.2014. 25258407 PMC4233285

[46] Zhong X, Cui P, Jiang J, Ning C, Liang B, Zhou J, Tian L, Zhang Y, Lei T, Zuo T, Ye L, Huang J, Chen H. 2021. Streptococcus, the predominant bacterium to predict the severity of liver injury in alcoholic liver disease. Front Cell Infect Microbiol 11:649060. doi:10.3389/fcimb.2021.649060. 33816353 PMC8010180

[47] Kale P, Khillan V, Sarin SK. 2018. Novel association of Streptococcus gallolyticus subspecies pasteurianus and hepatocelluar carcinoma: opening new frontiers. Scand J Gastroenterol 53:1354–1357. doi:10.1080/00365521.2018.1511826. 30332912

[48] Haruta I, Kikuchi K, Hashimoto E, Kato H, Hirota K, Kobayashi M, Miyake Y, Uchiyama T, Yagi J, Shiratori K. 2008. A possible role of histone-like DNA-binding protein of Streptococcus intermedius in the pathogenesis of bile duct damage in primary biliary cirrhosis. Clin Immunol 127:245–251. doi:10.1016/j.clim.2008.01.010. 18337173

[49] Castano-Rodriguez N, Goh KL, Fock KM, Mitchell HM, Kaakoush NO. 2017. Dysbiosis of the microbiome in gastric carcinogenesis. Sci Rep 7:15957. doi:10.1038/s41598-017-16289-2. 29162924 PMC5698432

[50] Ma X, Chi C, Fan L, Dong B, Shao X, Xie S, Li M, Xue W. 2019. The microbiome of prostate fluid is associated with prostate cancer. Front Microbiol 10:1664. doi:10.3389/fmicb.2019.01664. 31379800 PMC6659105

[51] Gao Z, Guo B, Gao R, Zhu Q, Qin H. 2015. Microbiota disbiosis is associated with colorectal cancer. Front Microbiol 6:20. doi:10.3389/fmicb.2015.00020. 25699023 PMC4313696

[52] Tsai WL, Chung RT. 2010. Viral hepatocarcinogenesis. Oncogene 29:2309–2324. doi:10.1038/onc.2010.36. 20228847 PMC3148694

[53] Gotland N, Uhre ML, Sandholdt H, Mejer N, Lundbo LF, Petersen A, Larsen AR, Benfield T. 2020. Increased risk of incident primary cancer after Staphylococcus aureus bacteremia: A matched cohort study. Medicine (Baltimore, MD) 99:e19984. doi:10.1097/MD.0000000000019984. PMC7220765 32332684

[54] Noguchi N, Fukuzawa M, Wajima T, Yokose K, Suzuki M, Nakaminami H, Kawai T, Moriyasu F, Sasatsu M. 2018. Specific clones of Staphylococcus lugdunensis may be associated with colon carcinoma. J Infect Public Health 11:39–42. doi:10.1016/j.jiph.2017.03.012. 28506660

[55] Van Beek M, Oravecz-Wilson KI, Delekta PC, Gu S, Li X, Jin X, Apel IJ, Konkle KS, Feng Y, Teitelbaum DH, Ruland J, McAllister-Lucas LM, Lucas PC. 2012. Bcl10 links saturated fat overnutrition with hepatocellular NF-kB activation and insulin resistance. Cell Rep 1:444–452. doi:10.1016/j.celrep.2012.04.006. 22708078 PMC3375919

[56] Broadfield LA, Goncalves Duarte JA, Schmieder R, Broekaert D, Veys K, Planque M, Vriens K, Karasawa Y, Napolitano F, Fujita S, Fujii M, Eto M, Holvoet B, Vangoitsenhoven R, Fernandez-Garcia J, Van Elsen J, Dehairs J, Zeng J, Dooley J, Alba Rubio R, van Pelt J, Grunewald TGP, Liston A, Mathieu C, Deroose CM, Swinnen JV, Lambrechts D, di Bernardo D, Kuroda S, De Bock K, Fendt SM. 2021. Fat induces glucose metabolism in non-transformed liver cells and promotes liver tumorigenesis. Cancer Res 81:1988–2001. doi:10.1158/0008-5472.CAN-20-1954. 33687947 PMC7611295

[57] Aizarani N, Saviano A, Mailly L, Durand S, Herman JS, Pessaux P, Baumert TF, Grün D. 2019. A human liver cell atlas reveals heterogeneity and epithelial progenitors. Nature 572:199–204. doi:10.1038/s41586-019-1373-2. 31292543 PMC6687507

[58] Chen S, Zhou Y, Chen Y, Gu J. 2018. fastp: an ultra-fast all-in-one FASTQ preprocessor. Bioinformatics 34:i884–i890. doi:10.1093/bioinformatics/bty560. 30423086 PMC6129281

[59] Magoc T, Salzberg SL. 2011. FLASH: fast length adjustment of short reads to improve genome assemblies. Bioinformatics 27:2957–2963. doi:10.1093/bioinformatics/btr507. 21903629 PMC3198573

[60] Edgar RC. 2013. UPARSE: highly accurate OTU sequences from microbial amplicon reads. Nat Methods 10:996–998. doi:10.1038/nmeth.2604. 23955772

[61] Wang Q, Garrity GM, Tiedje JM, Cole JR. 2007. Naive Bayesian classifier for rapid assignment of rRNA sequences into the new bacterial taxonomy. Appl Environ Microbiol 73:5261–5267. doi:10.1128/AEM.00062-07. 17586664 PMC1950982

