# Supplementary material for: The Intratumoral Bacterial Metataxonomic Signature of Hepatocellular Carcinoma
Source: Microbiol Spectr. 2022 Sep 29;10(5):e00983-22. doi: 10.1128/spectrum.00983-22 (PMC9602924; doi:10.1128/spectrum.00983-22)
Supplement: Supplemental file 4 — Supplemental material. Download spectrum.00983-22-s0004.pdf, PDF file, 6.1 MB [file spectrum.00983-22-s0004.pdf]

The intratumoral bacterial metataxonomic signature predicts hepatocellular carcinoma

Supplementary Material

Supplementary methods

Fresh tissue collection, preparation of paraffin sections, and hematoxylin-eosin (H&E) staining

Fresh cancer and peri-tumor tissues were collected during surgery with caution to avoid contamination. The fresh tissues were stored in sterile tubes cooled on ice immediately after being isolated from the patient’s body. All subsequent steps were performed on ice. All items were wiped clean with alcohol and autoclaved, including slides, knives, tubes, forceps, clippers, and containers. Each tissue was divided into three parts. One piece was processed immediately and used for tissue culture. Another part was fixed with paraformaldehyde (PFA) and used for H&E staining, Gram staining, and fluorescence in situ hybridization (FISH) analyses. The remaining part was stored at -80°C and used for PCR or other analysis. All these steps should be completed within one hour after the tissues were isolated from the body. To prepare paraffin sections, fresh surgery tissues were fixed overnight with 4% PFA. The tissues were trimmed and consecutively dehydrated using the following protocol: 4 h in 75% ethanol; 2 h in 85% ethanol; 2 h in 90% ethanol; 2 h in 95% ethanol; 1 h in 100% ethanol; 30 min in 100% ethanol; 30 min in ethanol/xylene (1:1); 5 – 10 min in 100% xylene; 5 – 10 min in 100% xylene. Next, the sections were socked in paraffin using the following steps: 5 – 10 min in melted paraffin at 65°C; 1 h in melted paraffin at 65°C; 1 h in melted paraffin at 65°C. The slides were embedded and allowed to cool at -20˚C. The paraffin-embedded tissues were cut into 4-μm sections. The sections were incubated overnight in a 56°C oven.

After deparaffinization, the sections were socked in 100% xylene for 20 min (two changes) and then dehydrated in 100% ethanol for 5 min (two changes) and 75% ethanol for 5 min. The sections were washed with running water. Next, the sections were stained with 2.5% hematoxylin solution for 3~5 min and then rinsed with running water. The sections were treated with 1% HCl in ethanol and washed with running water. The slides were washed with 1% ammonia solution for several seconds and were then rinsed using running water. Next, the sections were continuously dehydrated with 85% and 95% ethanol for 5min, respectively, staining and then stained with 1% eosin for 5 min. The sections were then consecutively dehydrated with 100% ethanol for 5 min (three changes) and 100% xylene for 5 min (two changes). Finally, the sections were mounted with neutral balsam.

Gram staining of HCC or peri-tumor tissues

Gram staining of tissues was performed with the Gram Staining kit (Wuhan Servicebio Technology Co., Ltd. Wuhan, Hu Bei, China) according to the manufacturer's instructions. Briefly, paraffin sections were deparaffinized using the procedure: 20 min in xylene (two changes), 5 min in 100% ethanol (two changes), 5 min in 75% alcohol, and washed with tap water. sections were stained with Gram Staining Solution A (2.5% crystal violet/ammonium oxalate in ethanol) for 10 s - 30 s, washing, and then washed with distilled water. slides were stained with Gram Staining Solution B (1% iodine) for 1 min and then washed with distilled water. The Gram Staining Solution C (95% ethanol) was used to rinse the slides from the frosted surface side of the slides for differentiation until the solution running off the section was colorless. The sections were immersed in Gram Staining Solution D (2.5% safranin) for one second and then washed with distilled water. The sections were dried in

an oven at 60˚C. The dried sections were quickly immersed in 100% ethanol (three changes of 1s, 3s, and 5s in sequence) for dehydration. The sections were immersed in clean xylene for 5 min to increase transparency and then sealed with neutral balsam. The slides were scanned using the NanoZoomer S60 Digital Slide Scanner (C13210, Hamamatsu Photonics K.K. Co., Ltd., Japan). The images were viewed with NDP.view version 2.7.43 (Hamamatsu Photonics K.K. Co., Ltd., Japan).

16S rRNA fluorescence in situ hybridization (FISH)

Formalin-fixed paraffin-embedded (FFPE) tissue slides were deparaffinized and rehydrated using the procedure: 15 min in 100% xylene (two changes), 5 min in 100% ethanol (two changes), 5 min in 85% ethanol, and 5 min in 75% ethanol. The sections were washed with diethylpyrocarbonate (DEPC)-treated water. Slides were incubated for 10~15 min in boiled citrate-EDTA antigen retrieval solution and the solution was let cool naturally. Sections were incubated in prehybridization solution at 37˚C for one hour. Hybridization was performed overnight at 37˚C using Cy3-labelled probes EUB338 5'-GCTGCCTCCCGTAGGAGT-3' and Cy5-labelled non-specific complement probe 5'-CGACGGAGGGCATCCTCA-3' (5 ng/μL) as reported by Nejman D., *et al* (1). Sections were washed with 2 × SSC at 37˚C for 10 min, 1 × SSC at 37˚C for 5 min (two changes), and 0.5 × SSC at room temperature for 10 min. The nucleus was counterstained with DAPI (2 μg/μL) for 8 min in a dark place. Sections were mounted with antifade Mountant. The signal was captured using the PANNORAMIC MIDI digital slide scanner (3DHISTECH Ltd. Budapest, Hungary). The images were viewed with CaseViewer version 2.4 (3DHISTECH Ltd. Budapest, Hungary).

Aerobic and anaerobic cultures of cancer or peri-tumor tissues

Fresh cancer and peri-tumor tissues were immersed in normal saline in a sterile culturing dish. The surgical blade was wiped with ethanol and passed through a flame. After cooling down, the blade was used to cut the tissues into two pieces, which were then put into two 1.5-mL Eppendorf tubes. One part of the tissues was subjected to aerobic culture using the Brain Heart Infusion (BHI) Broth (HB8297-5, Hopebio Biotechnology Co., LTD., Qingdao, Shandong, China). Another part of the tissues was used for anaerobic culture using Gifu Anaerobic Medium (GRM) (HB8518-1, Hopebio Biotechnology Co., LTD., Qingdao, Shandong, China) (supplemented with 0.0005% vitamin k1 and 25 μg/mL hemin). The tissue in each tube was ground for ~250 seconds in a freezing grinder (4˚C, 2500 rpm) using a pre-autoclaved stainless-steel bead (6.2 mm in diameter) (Shanghai Jingxin Industrial Development Co. LTD, Shanghai, China). During the grinding process, the tubes were examined several times to ensure that the tissue was completely homogenized. A 150-μL homogenate of each sample was pipetted to an agar plate (1.5% agar in GAM or BHI medium) and spread on the surface with a sterilized triangle end glass spreader. For aerobic culturing, the plates were incubated in an incubator and cultured at 37˚C for 3~5 days. For anaerobic culture, the plates were placed in a 7.0 L sealed culture tank (Mitsubishi Gas Chemical Company, INC. Japan) was equipped with three 2.5 L anaerobic gas-producing bags (HBYY001, Hopebio Biotechnology Co., LTD., Qingdao, Shandong, China) and oxygen indicator (HBYY004, Hopebio Biotechnology Co., LTD., Qingdao, Shandong, China). The sealed culture tank was placed in an incubator at 37˚C for 3~6 days. The negative controls for aerobic and anaerobic cultures were blank BHI and GAM

media, respectively. To prepare positive controls for aerobic culture, we collected the environmental microbiota by wiping a lab door handle with a sterile cotton swab, and the cotton swab was immersed in the BHI medium used for aerobic culture. The positive control for anaerobic culturing was done in the same way except for the use of the GAM medium.

Taxonomic identification of bacteria colony isolated from the tissue culturing cultures

The colonies from the aerobic and anaerobic cultures of cancer or peri-tumor tissues were identified by 16S rRNA gene sequencing anaerobic using the 2 × EasyTaq® PCR SuperMix (TransGen Biotech Co., LTD., Beijing, China). Aerobic colonies were picked and amplified by agitation overnight at 37˚C in BHI media, while anaerobic colonies were picked and cultured overnight at 37˚C in GAM media without agitation. 10 μL of the culture was diluted with 90 μL of H_2O. The PCR reaction mix contained 2 μL of diluted DNA template, 1 μL of each primer (forward primer 27F, 5'-AGAGTTTGATCCTGGCTCAG-3' and reverse primer 1492R, 5'-GGTTACCTTGTTACGACTT-3'), 10 μL of 2 × EasyTaq PCR SuperMIX and 6 μL of H_2O. The PCR amplification cycle consisted of a pre-denaturing step at 94°C for 5 min, followed by 30 cycles of 94°C for 30 s, 53°C for 30 s, 72°C for 2 min, and a final extension step at 72°C for 2 min. The PCR products were analyzed using agarose electrophoresis followed by Sanger DNA sequencing using the same primer pairs. The taxonomic identities of the sequences were determined by blasting the NT database using the BLASTN program in GenBank (http://www.ncbi.nlm.nih.gov). The PCR sequences, taxonomic identities, and the BLAST results were deposited in **Supplementary Table S2**.

Quantitative PCR (qPCR) validation of 16S rRNA gene of selected taxa

Total cellular DNA was extracted from liver tissues as described above. qPCR was performed with BeyoFast Probe qPCR Mix (2X, Low ROX) (Beyotime, Shanghai, China) using the Roche LightCycler® 480 II instrument (Roche Diagnostics Corporation, Indianapolis, USA) according to the manufacturer's instructions. The primers Gamma395f (5'-CMATGCCGCGTGTGTGAA-3') and Gamma871r (5'-ACTCCCCAGGCGGTCDACTTA-3') for Gammaproteobacteria were used in the qPCR analysis, as described by Pfeiffer S, et al (2). The TestPrime 1.0 program (https://www.arb-silva.de/search/testprime/) was used to evaluate the specificity of this primer pair on the SILVA database. The results indicate that this primer pair targets 28.4% of Gammaproteobacteria in the SILVA database. A qPCR analysis of human albumin exon 12 was performed in parallel using the primer pair: forward-TGTTGCATGAGAAAACGCCA and reverse-GTCGCCTGTTCAACCAAGGAT (3). The PCR reaction mix contains 0.5 μL diluted DNA template (100 ng/μL), 1 μL of each primer (final concentration 1 nmol), 5 μL 2 × BeyoFast Probe qPCR Mix (2X, Low ROX), and 2.5 μL H_2O. The three-step qPCR amplification cycle consists of a pre-denaturing step at 95°C for 10 min, followed by 42 cycles of 95°C for 5 s, 55°C for 10 s, and a final extension step at 72°C for 30 s. The melting curve was generated using the same conditions as above. The analysis was performed in a 384-well plate and 10 negative controls using blank reagents (no DNA template) were analyzed in parallel on each plate. The fluorescence cycle threshold (Ct) values were recorded for each amplicon. Delta Ct (ΔCt) method was used to normalize the expression of 16S rRNA gene (Ct(16S rRNA) – Ct

(albumin)). The ΔCt values were log2-transformed and the p-value was calculated using a two-sided, unpaired Student's *t*-test.

Random-forest machine learning

The random-forest machine learning model was built based on OTU or Class. A training cohort containing 75% of total cases was built with samples randomly selected from the normal and HCC samples using the R "sample" function. The remaining 25% of samples were used as the validation cohort. Random-forest machine learning analysis was performed using R package randomForest v4.6.14 and 500 trees were built with 10-fold cross-validation. The normalized additive predicting probability was calculated and used as the final predicting probability. The higher probability of the binary classification was used as the predictive label. The features were ranked from high to low Mean Decrease Accuracy value and selected based on the cross-validation curve. The top-5 features were selected to build the model. The prediction power of the selected features was validated in the validation cohort.

Chi-square test

The association between taxon abundance and the clinicopathological parameters of HCC patients was analyzed using the Chi-square test. Taxon abundance was determined based on the median read count of each taxon. Taxon with an abundance lower than the median was classified as "low", while taxon with an abundance higher than the median was classified as "high". A two-sided Chi-square test was performed using GraphPad Prism 7.04. $p < 0.05$ was considered as statistically significant.

Statistics

Statistical significance was calculated using a two-sided Student's test. P-value or corrected p-value < 0.05 is considered as statistically significant. All measured values were expressed as mean ± SE.

Figure S1

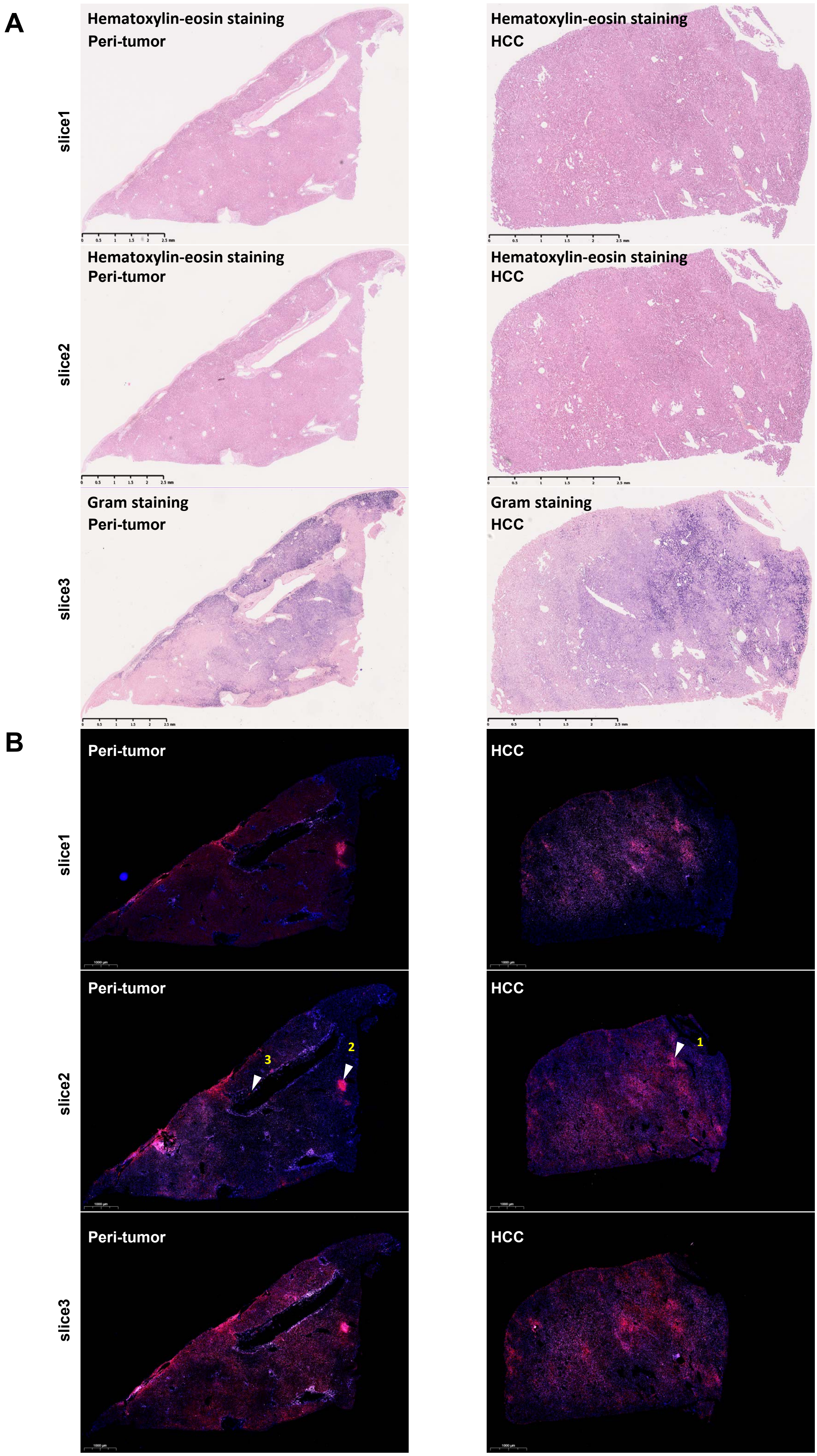
A
Hematoxylin-eosin staining
Peri-tumor
Hematoxylin-eosin staining
HCC
slice1
Hematoxylin-eosin staining
Peri-tumor
Hematoxylin-eosin staining
HCC
slice2
Gram staining
Peri-tumor
Gram staining
HCC
slice3
B
Peri-tumor
HCC
slice1
Peri-tumor
3
2
HCC
1
slice2
Peri-tumor
HCC
slice3

Figure S1. 16S rRNA fluorescence in situ hybridization (FISH) analysis of an HCC tissue and its associated peri-tumor tissue. (**A**) Three pieces of sequential formalin-fixed paraffin-embedded (FFPE) sections of peri-tumor (left) or liver cancer (right) were deparaffinized, rehydrated, and stained with hematoxylin-eosin (H&E, panel 1 and panel 2) or Gram staining (panel 3). (**B**) Three pieces of sequential FFPE liver cancer sections were deparaffinized, rehydrated, and probed with Cy3-labelled probes EUB338 (5'-GCTGCCTCCCGTAGGAGT-3') (red color) or Cy5-labelled non-specific complement probe (5'-CGACGGAGGGCATCCTCA-3') (pink color) Both probes have been recently used to analyze the microbiota of human cancer tissues (1). The nuclei were counterstained with diamidino-phenyl-indole (DAPI). The white arrowheads indicate the regions highlighted in Fig. 1 and Figure S2, Figure S3 (region 2), and Figure S4 (region 3).

Figure S2

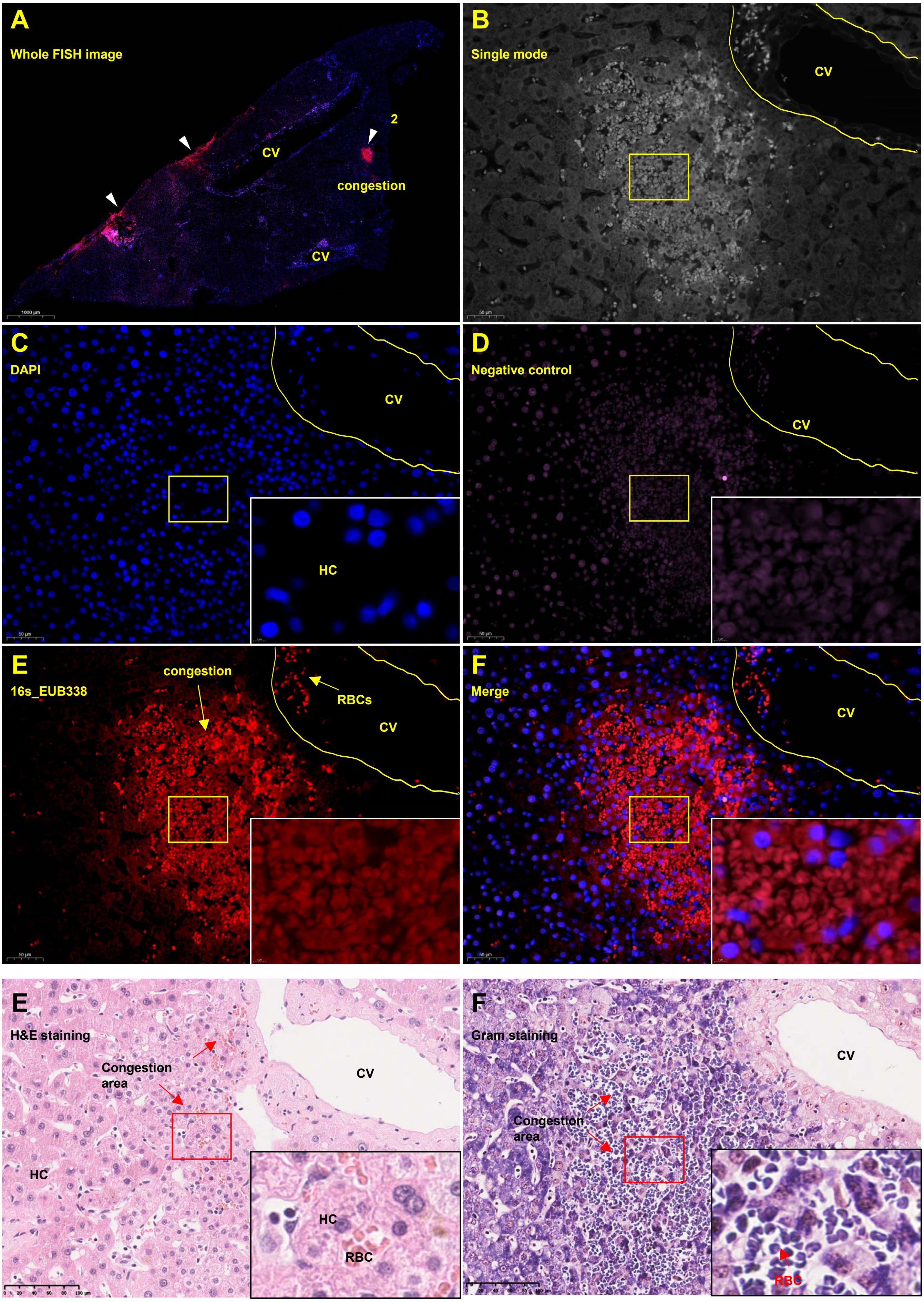
A
Whole FISH image
CV
2
congestion
CV
B
Single mode
CV
C
DAPI
CV
HC
D
Negative control
CV
E
16s_EUB338
congestion
RBCs
CV
F
Merge
CV
E
H&E staining
Congestion area
CV
HC
HC
RBC
F
Gram staining
CV
Congestion area
RBC

Figure S2. FISH analysis of a peri-tumor tissue revealing accumulation of RBCs in the congestion area. (**A**) The overview of the analyzed region. The white arrowheads indicate the congestion area. CV, central vein. (**B**) The single-mode of region 2 is labeled in (**A**). The CV area was illustrated with the yellow curve. The yellow box indicated the region that was enlarged. (**C**) DAPI staining. The region of the yellow box was enlarged in the lower right corner. HC, hepatocyte. (**D**) Staining of the complement probe. (**E**) EUB338 staining. The extensive staining was concentrated in the congestion region. The yellow arrows indicate the clusters of RBCs in the congestion area. (**F**) The merged image of different staining. (**E**) The H&E staining image. The red arrows indicate the RBCs in the congestion area. The red boxes indicate the same region as highlighted in the lower right corner. (**F**) The Gram staining image of a sequential tissue section of the corresponding region. Since the sections used for FISH and H&E staining were sequential sections, they could not be 100% merged to show the overlapping due to small changes between the sections.

Figure S3

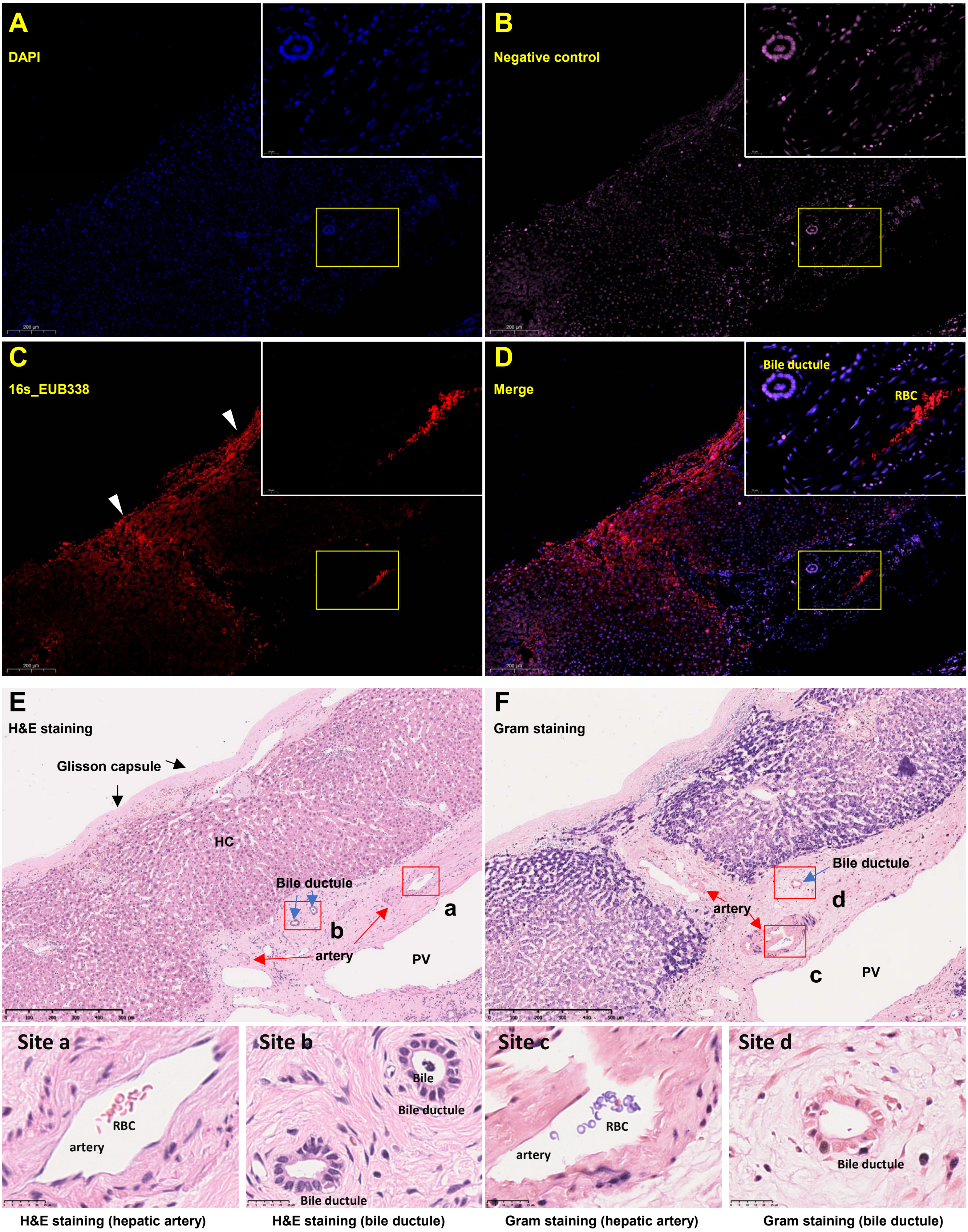

A
DAPI
B
Negative control
C
16s_EUB338
D
Merge
Bile ductule
RBC
E
H&E staining
Glisson capsule
HC
Bile ductule
b
a
artery
PV
F
Gram staining
Bile ductule
d
artery
c
PV
Site a
RBC
artery
Site b
Bile
Bile ductule
Bile ductule
Site c
RBC
artery
Site d
Bile ductule
H&E staining (hepatic artery)
H&E staining (bile ductule)
Gram staining (hepatic artery)
Gram staining (bile ductule)

Figure S3. FISH staining of the peri-tumor tissue shows negative staining of the bile duct. (**A**) DAPI staining. The yellow boxes indicated the region enlarged in the top right corner. (**B**) Staining of the complement probe. (**C**) The EUB338 staining. The white arrowheads indicated the region of congestion. (**D**) The merged image of different staining. RBC, red blood cell. The magnified FISH image showed strong staining of RBCs in the artery and negative staining of bile ductule cells. (**E**) H&E staining. The black arrows indicate the Glisson capsule. The blue arrows indicate the bile ductule. The red arrows indicate the artery. The red boxes indicated the sites (a, b,) enlarged in the bottom panel. The magnified H&E staining images showed the artery (bottom left) or bile ductule (bottom right). HC, hepatocyte. PV, portal vein. (**F**) Gram staining image of a sequential tissue section of the corresponding region. The bile and the bile duct cells were negatively stained, compared with the intensive staining of RBCs in the artery lumen. The bottom panel shows the magnified Gram staining images of the artery and bile ductule (sites c and d). The RBCs in the artery lumen were stained blue-violet.

Figure S4

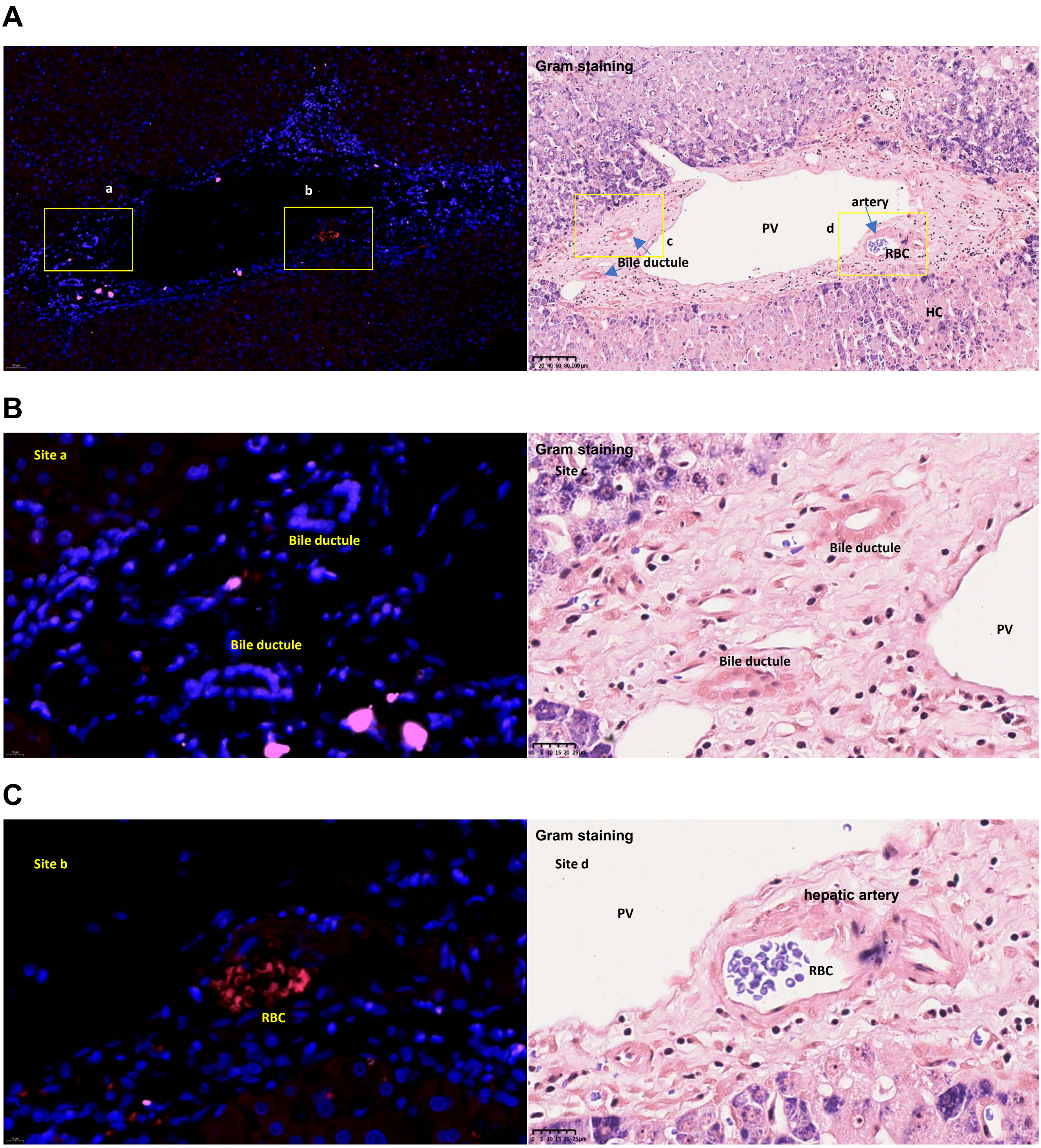
A
a
b
Gram staining
artery
PV
d
c
Bile ductule
RBC
HC
B
Site a
Bile ductule
Bile ductule
Gram staining
Site c
Bile ductule
PV
Bile ductule
C
Site b
RBC
Gram staining
Site d
PV
hepatic artery
RBC

Figure S4. RBCs in the artery were positively stained, while bile ductules were negatively stained by a 16S rRNA FISH probe. (**A**) The merged image of EUB338, complement probe, and DAPI staining (left) and the image of Gram staining of the same region. Yellow boxes a and b were enlarged in (**B**) and (**C**), respectively. RBC, red blood cell. PV, portal vein. HC, hepatocytes. (**B**) The regions highlighted by yellow box a were enlarged. (**C**) The region highlighted by yellow box b was enlarged.

Figure S5

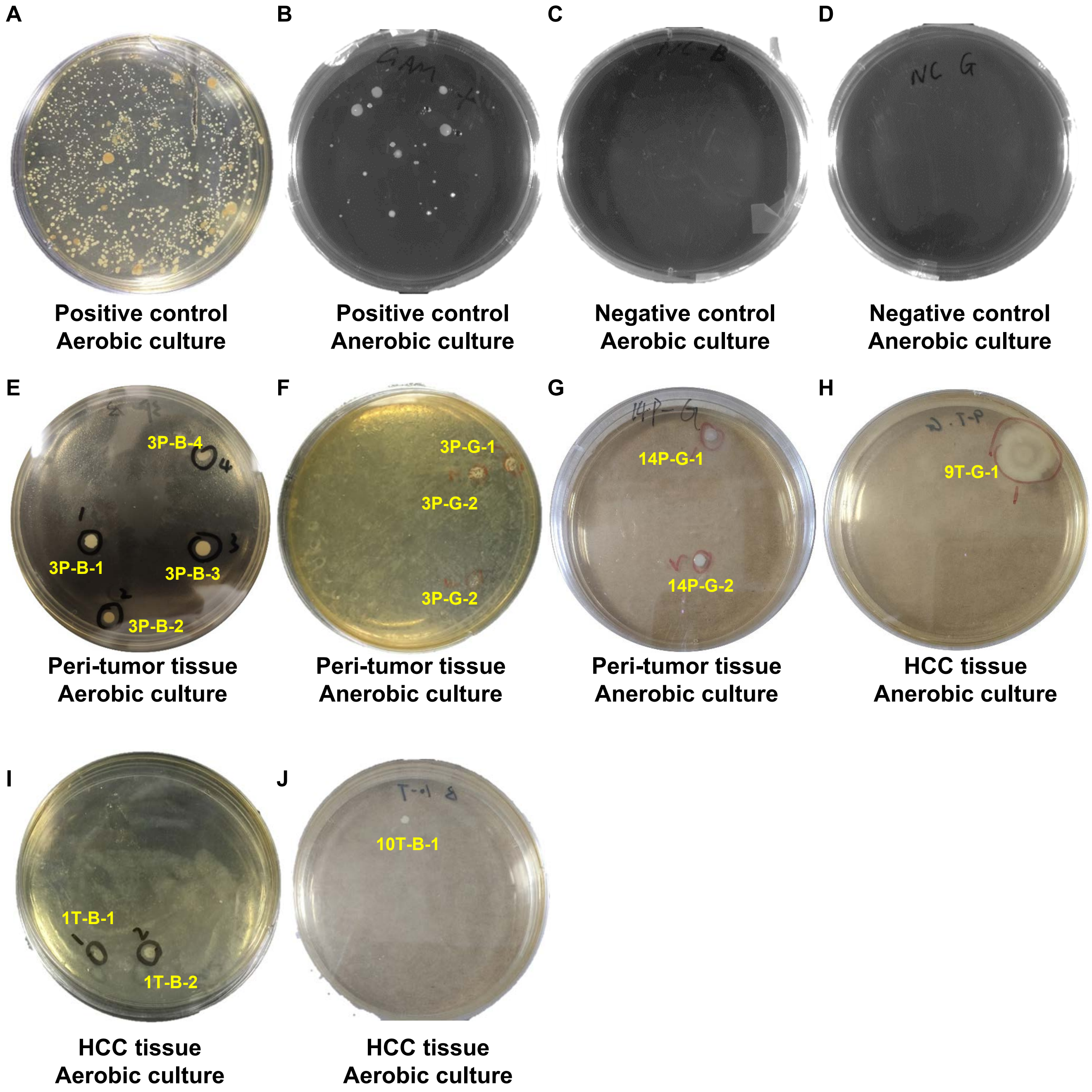

A
Positive control
Aerobic culture
B
Positive control
Anerobic culture
C
Negative control
Aerobic culture
D
Negative control
Anerobic culture
E
3P-B-4
3P-B-1
3P-B-3
3P-B-2
Peri-tumor tissue
Aerobic culture
F
3P-G-1
3P-G-2
3P-G-2
Peri-tumor tissue
Anerobic culture
G
14P-G-1
14P-G-2
Peri-tumor tissue
Anerobic culture
H
9T-G-1
HCC tissue
Anerobic culture
I
1T-B-1
1T-B-2
HCC tissue
Aerobic culture
J
10T-B-1
HCC tissue
Aerobic culture

Figure S5. Viable bacteria were cultured from freshly prepared homogenates of liver cancer or peri-tumor tissues. (**A**) Positive control for aerobic culturing. The environmental microbiota was collected from the door handle using a dry cotton swab. The dish was incubated at 37°C. The picture was captured regularly on the desktop. (**B**) Positive control of anerobic culturing. The dish was incubated at 37°C in a container used for anaerobic culturing. The picture was captured using the Fujifilm LAS3000 system. (**C**) and (**D**) The negative controls of aerobic or anaerobic culture using blank BHI or GAM medium. (**E**) and (**F**) Aerobic or anaerobic culturing of a peri-tumor tissue (3P). The labeled colonies were subjected to sub-culturing, DNA extraction, and DNA sequencing using the primers targeting the V3-V4 variable region of bacteria 16S rRNA gene. The tissue debris was left on the plate. The bacteria colonies were picked up with sterilized toothpicks for sub-culturing. (**G**) Anaerobic culturing of the peri-tumor tissue (14P). (**H**)-(**J**) Aerobic culturing of HCC tissues 9T, 1T, and 10T. The tissue debris was spread on the plate.

Figure S6

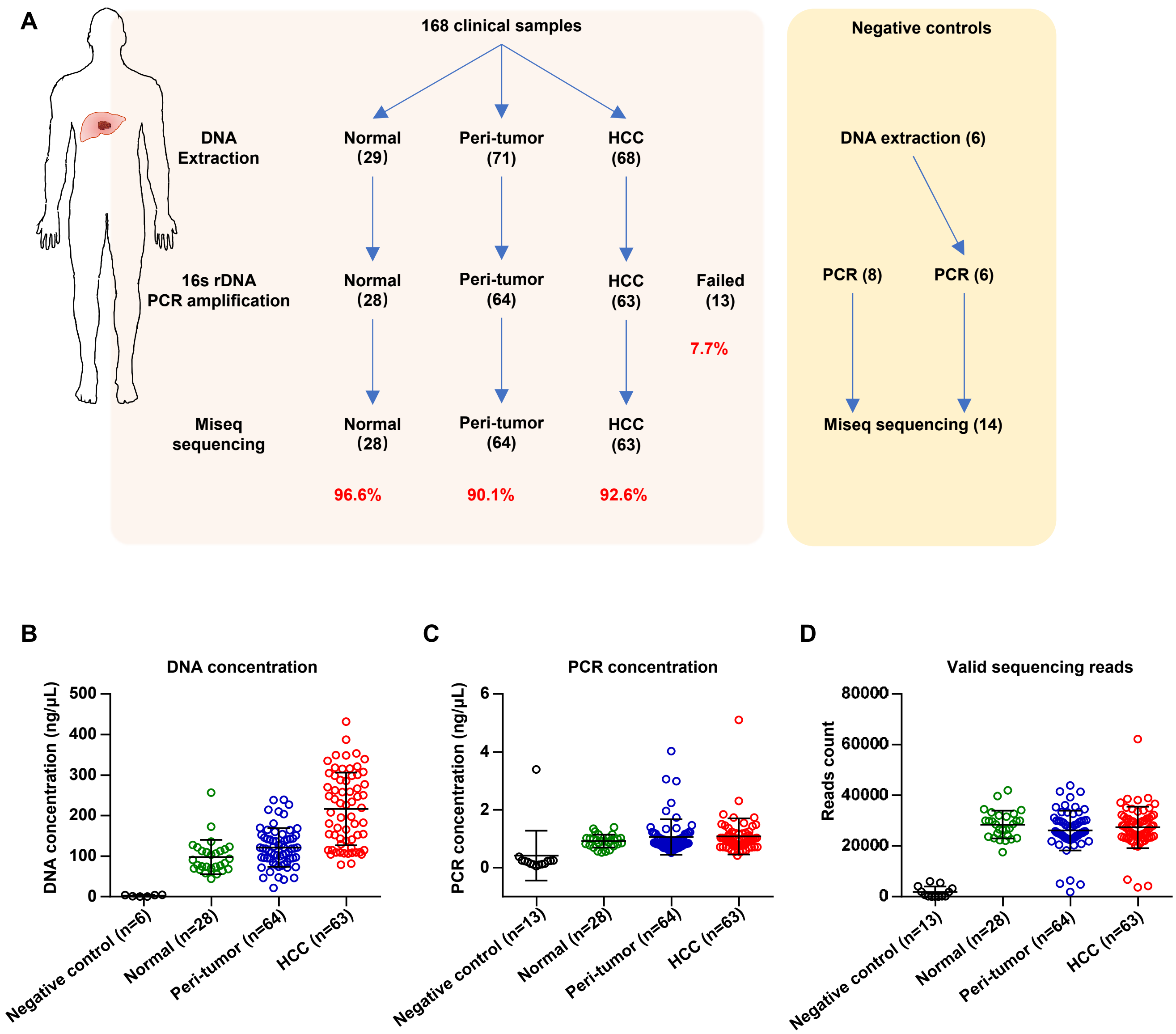
A
168 clinical samples
DNA Extraction
Normal (29)
Peri-tumor (71)
HCC (68)
16s rDNA PCR amplification
Normal (28)
Peri-tumor (64)
HCC (63)
Failed (13)
7.7%
Miseq sequencing
Normal (28)
Peri-tumor (64)
HCC (63)
96.6%
90.1%
92.6%
Negative controls
DNA extraction (6)
PCR (8)
PCR (6)
Miseq sequencing (14)
B
DNA concentration
DNA concentration (ng/μL)
Negative control (n=6)
Normal (n=28)
Peri-tumor (n=64)
HCC (n=63)
C
PCR concentration
PCR concentration (ng/μL)
Negative control (n=13)
Normal (n=28)
Peri-tumor (n=64)
HCC (n=63)
D
Valid sequencing reads
Reads count
Negative control (n=13)
Normal (n=28)
Peri-tumor (n=64)
HCC (n=63)

Figure S6. 16S rRNA sequencing of 168 normal, peri-tumor, and HCC tissues. (**A**) Flowchart of the metataxonomic analysis of 168 liver tissues. Thirteen samples, including one normal, 7 peri-tumor, and 5 HCC tissues, failed in 16S rRNA gene PCR amplification. Negative control analyses of blank reagents (DNA extraction and PCR experiments) were also performed in parallel. Fourteen samples were subjected to Miseq sequencing. (**B**) DNA concentration (μg/μL) of the liver samples. (**C**) PCR products were quantified before being used for Miseq sequencing. (**D**) Valid reads of liver samples after filtering.

Figure S7

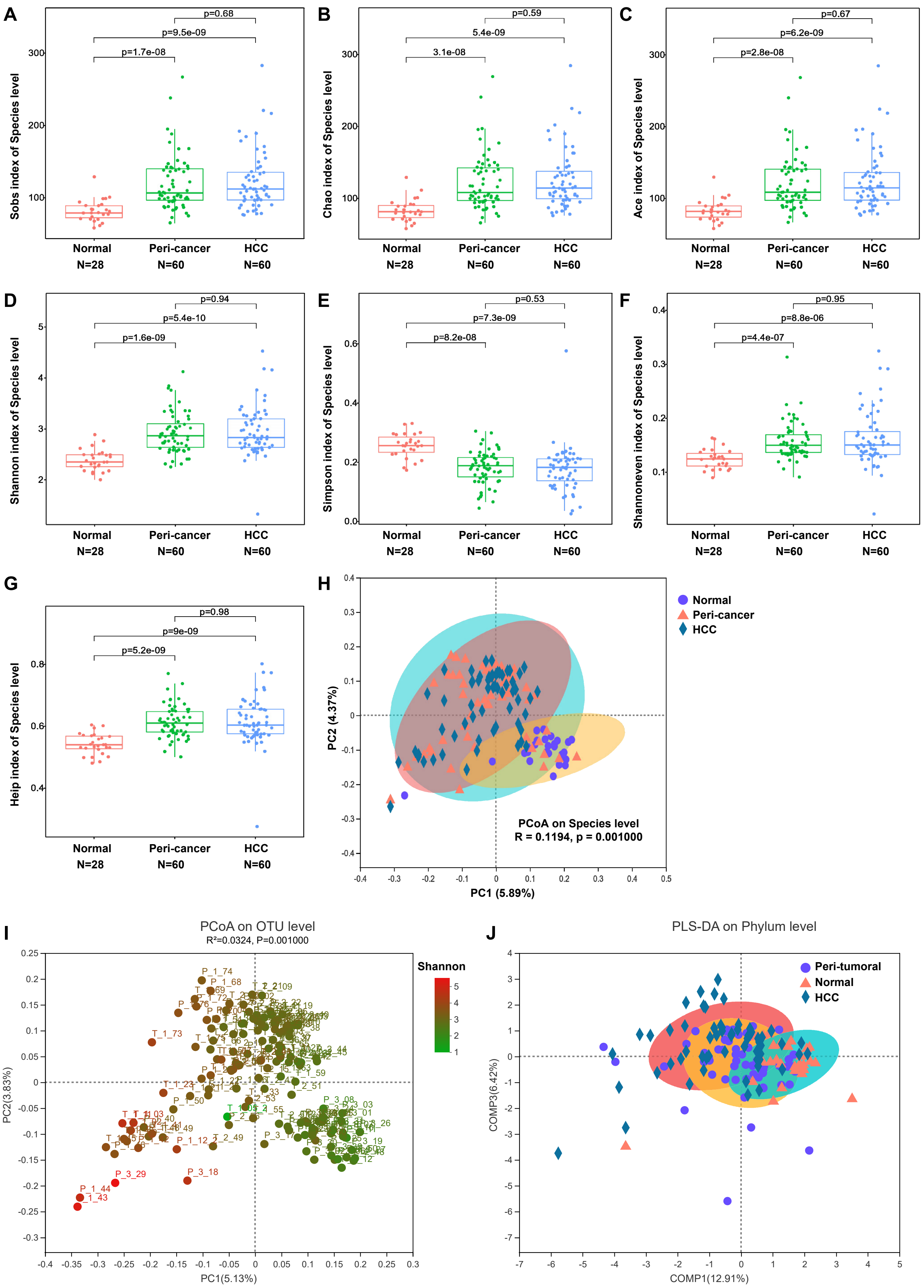
A
Sobs index of Species level
p=0.68
p=9.5e-09
p=1.7e-08
B
Chao index of Species level
p=0.59
5.4e-09
3.1e-08
C
Ace index of Species level
p=0.67
p=6.2e-09
p=2.8e-08
D
Shannon index of Species level
p=0.94
p=5.4e-10
p=1.6e-09
E
Simpson index of Species level
p=0.53
p=7.3e-09
p=8.2e-08
F
Shannoneven index of Species level
p=0.95
p=8.8e-06
p=4.4e-07
G
Heip index of Species level
p=0.98
p=9e-09
p=5.2e-09
Normal
N=28
Peri-cancer
N=60
HCC
N=60
H
PC2 (4.37%)
PC1 (5.89%)
Normal
Peri-cancer
HCC
PCoA on Species level
R = 0.1194, p = 0.001000
I
PCoA on OTU level
R²=0.0324, P=0.001000
PC2(3.83%)
PC1(5.13%)
Shannon
J
PLS-DA on Phylum level
Peri-tumoral
Normal
HCC
COMP3(6.42%)
COMP1(12.91%)

Figure S7. Alpha diversity of different liver microbiota at the species level. (**A**)-(**C**) Richness indexes Sobs, Chao, and Ace were significantly increased in peri-tumor and HCC microhabitats. N indicates the sample number. (**D**) and (**E**) Diversity index Shannon was significantly increased while Simpson was significantly decreased in peri-tumor and HCC microhabitats compared with normal microhabitats. (**F**) and (**G**) Evenness indexes Shannoneven and Heip were significantly increased in peri-tumor and HCC microhabitats compared with normal microhabitats. (**H**) PCoA analysis on Species-level of normal, peri-tumor, and HCC subjects. The difference between the group was tested using the Adonis algorithm and the number of replacements was 999. (**I**) PCoA analysis on OTU level of normal, peri-tumor, and HCC microbiota. The samples were colored based on the Shannon index. The difference between the group was tested using the Adonis algorithm and the number of replacements was 999. (**J**) PLS-DA analysis revealed specific bacterial taxa which contribute to the major differences between different microhabitats. The ellipses indicated the confidence interval.

Figure S8

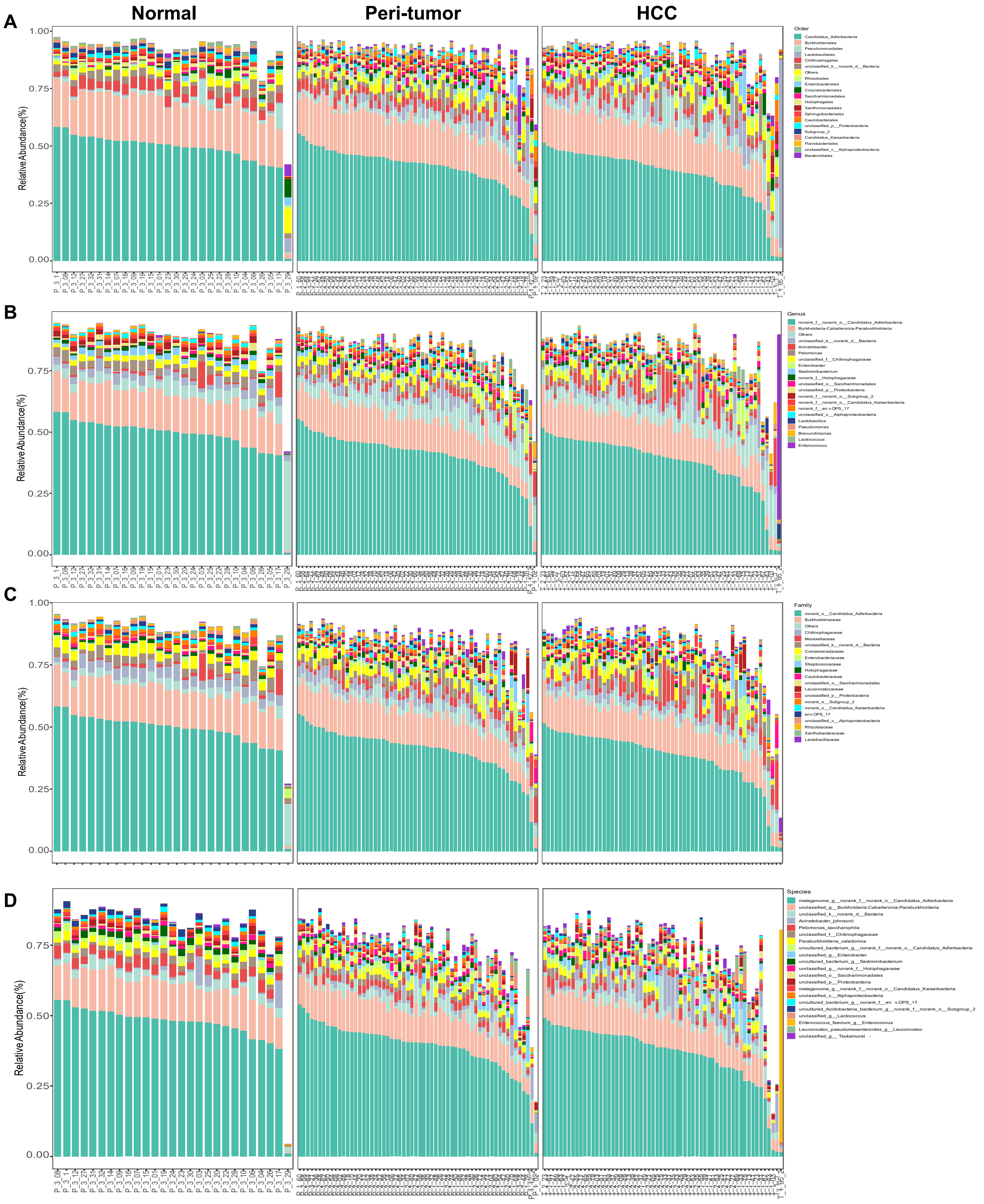
A
Normal
Peri-tumor
HCC
Relative Abundance(%)
1.00
0.75
0.50
0.25
0.00
Order
Candidatus_Adlerbacteria
Burkholderiales
Pseudomonadales
Lactobacillales
Chitinophagales
unclassified_k__norank_d__Bacteria
Others
Rhizobiales
Enterobacterales
Corynebacteriales
Saccharimonadales
Holophagales
Xanthomonadales
Sphingobacteriales
Caulobacterales
unclassified_p__Proteobacteria
Subgroup_2
Candidatus_Kaiserbacteria
Flavobacteriales
unclassified_c__Alphaproteobacteria
Bacteroidales
B
Genus
norank_f__norank_o__Candidatus_Adlerbacteria
Burkholderia-Caballeronia-Paraburkholderia
Others
unclassified_k__norank_d__Bacteria
Acinetobacter
Pelomonas
unclassified_f__Chitinophagaceae
Enterobacter
Sediminibacterium
norank_f__Holophagaceae
unclassified_o__Saccharimonadales
unclassified_p__Proteobacteria
norank_f__norank_o__Subgroup_2
norank_f__norank_o__Candidatus_Kaiserbacteria
norank_f__en v.OPS_17
unclassified_c__Alphaproteobacteria
Lactobacillus
Pseudomonas
Brevundimonas
Lactococcus
Enterococcus
C
Family
norank_o__Candidatus_Adlerbacteria
Burkholderiaceae
Others
Chitinophagaceae
Moraxellaceae
unclassified_k__norank_d__Bacteria
Comamonadaceae
Enterobacteriaceae
Streptococcaceae
Holophagaceae
Caulobacteraceae
unclassified_o__Saccharimonadales
Leuconostocaceae
unclassified_p__Proteobacteria
norank_o__Subgroup_2
norank_o__Candidatus_Kaiserbacteria
env.OPS_17
unclassified_c__Alphaproteobacteria
Rhizobiaceae
Xanthobacteraceae
Lactobacillaceae
D
Species
metagenome_g__norank_f__norank_o__Candidatus_Adlerbacteria
unclassified_g__Burkholderia-Caballeronia-Paraburkholderia
unclassified_k__norank_d__Bacteria
Acinetobacter_johnsonii
Pelomonas_saccharophila
unclassified_f__Chitinophagaceae
Paraburkholderia_caledonica
uncultured_bacterium_g__norank_f__norank_o__Candidatus_Adlerbacteria
unclassified_g__Enterobacter
uncultured_bacterium_g__Sediminibacterium
unclassified_g__norank_f__Holophagaceae
unclassified_o__Saccharimonadales
unclassified_p__Proteobacteria
metagenome_g__norank_f__norank_o__Candidatus_Kaiserbacteria
unclassified_c__Alphaproteobacteria
uncultured_bacterium_g__norank_f__en v.OPS_17
uncultured_Acidobacteria_bacterium_g__norank_f__norank_o__Subgroup_2
unclassified_g__Lactococcus
Enterococcus_faecium_g__Enterococcus
Leuconostoc_pseudomesenteroides_g__Leuconostoc
unclassified_g__ Tsukamurel -

Figure S8. The structure and abundance of the microbiota in normal, peri-tumor, and HCC microhabitats. The structure and relative abundance of the normal, peri-tumor, and HCC microbiota at Order (**A**), Family (**B**), Genus (**C**), and Species (**D**) levels using OTU data.

Figure S9

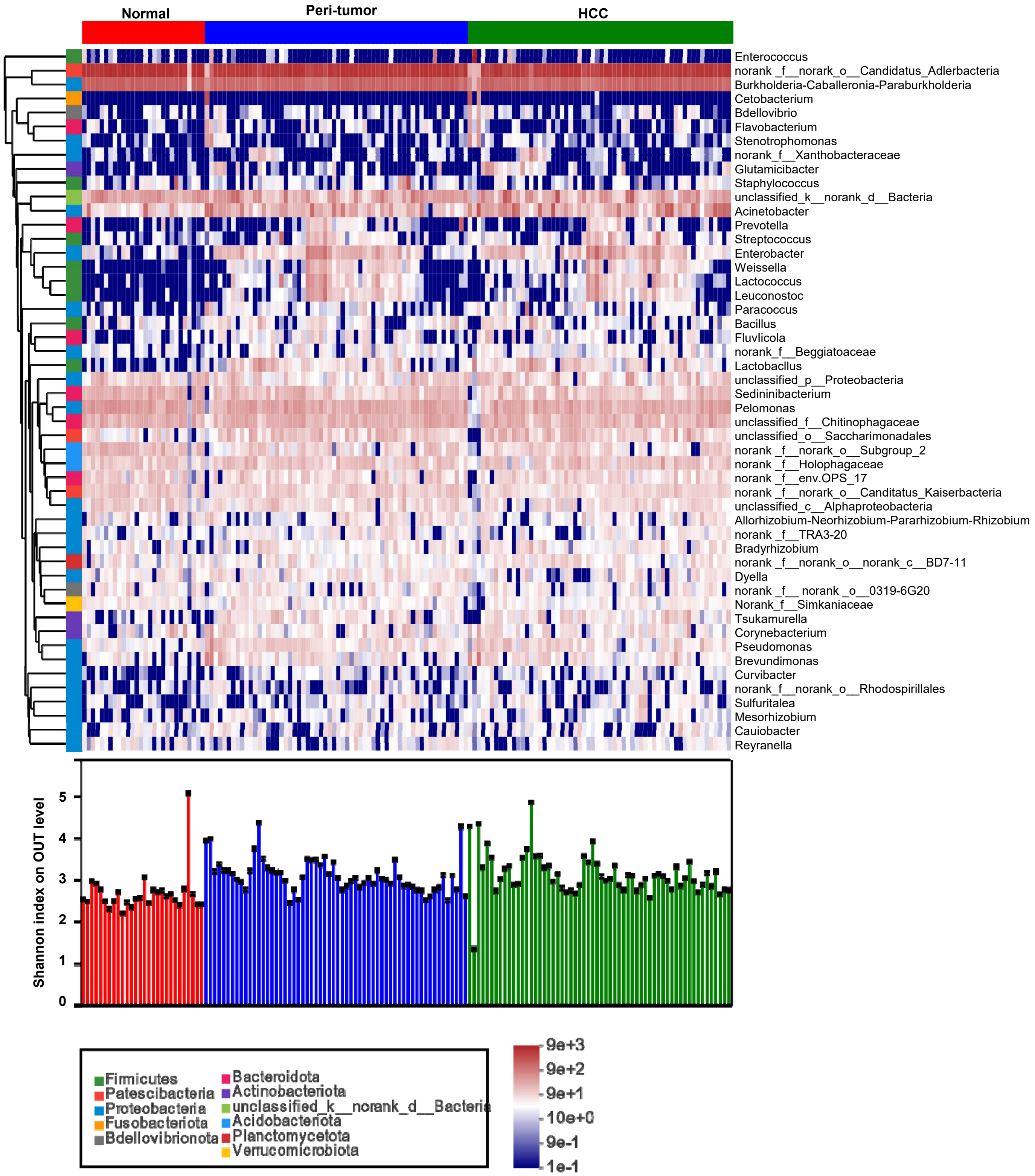

Normal
Peri-tumor
HCC
Enterococcus
norank _f__norark_o__Candidatus_Adlerbacteria
Burkholderia-Caballeronia-Paraburkholderia
Cetobacterium
Bdellovibrio
Flavobacterium
Stenotrophomonas
norank_f__Xanthobacteraceae
Glutamicibacter
Staphylococcus
unclassified_k__norank_d__Bacteria
Acinetobacter
Prevotella
Streptococcus
Enterobacter
Weissella
Lactococcus
Leuconostoc
Paracoccus
Bacillus
Fluvlicola
norank_f__Beggiatoaceae
Lactobacllus
unclassified_p__Proteobacteria
Sedininibacterium
Pelomonas
unclassified_f__Chitinophagaceae
unclassified_o__Saccharimonadales
norank _f__norark_o__Subgroup_2
norank _f__Holophagaceae
norank _f__env.OPS_17
norank _f__norark_o__Canditatus_Kaiserbacteria
unclassified_c__Alphaproteobacteria
Allorhizobium-Neorhizobium-Pararhizobium-Rhizobium
norank _f__TRA3-20
Bradyrhizobium
norank _f__norank_o__norank_c__BD7-11
Dyella
norank _f__ norank _o__0319-6G20
Norank_f__Simkaniaceae
Tsukamurella
Corynebacterium
Pseudomonas
Brevundimonas
Curvibacter
norank_f__norank_o__Rhodospirillales
Sulfuritalea
Mesorhizobium
Cauiobacter
Reyranella
Shannon index on OUT level
0
1
2
3
4
5
Firmicutes
Patescibacteria
Proteobacteria
Fusobacteriota
Bdellovibrionota
Bacteroidota
Actinobacteriota
unclassified_k__norank_d__Bacteria
Acidobacteriota
Planctomycetota
Verrucomicrobiota
9e+3
9e+2
9e+1
10e+0
9e-1
1e-1

Figure S9. Hierarchical clustering of taxa at Genus level across different liver microbiota. Heatmap analysis revealed the relationship between identified Genera and the abundance across different liver microbiota. The Phyla of each Genus name was illustrated on the right side of the heatmap. The Shannon index on the OTU level was displayed at the bottom of the heatmap. The heatmap was generated using the Vegan package of R.

Figure S10

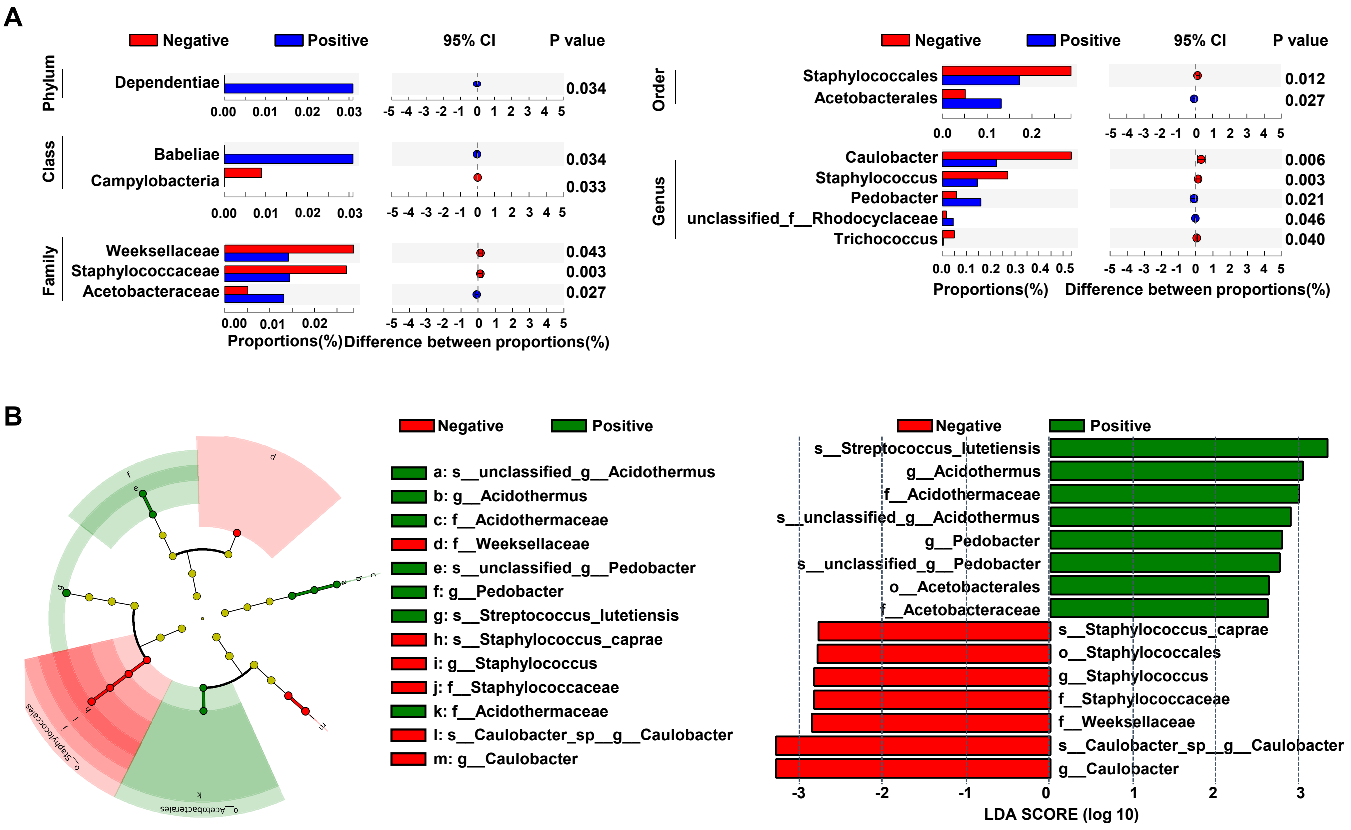
A
Negative
Positive
95% CI
P value
Phylum
Dependentiae
0.034
Class
Babeliae
Campylobacteria
0.034
0.033
Family
Weeksellaceae
Staphylococcaceae
Acetobacteraceae
0.043
0.003
0.027
Proportions(%)
Difference between proportions(%)
Order
Staphylococcales
Acetobacterales
0.012
0.027
Genus
Caulobacter
Staphylococcus
Pedobacter
unclassified_f__Rhodocyclaceae
Trichococcus
0.006
0.003
0.021
0.046
0.040
B
a: s__unclassified_g__Acidothermus
b: g__Acidothermus
c: f__Acidothermaceae
d: f__Weeksellaceae
e: s__unclassified_g__Pedobacter
f: g__Pedobacter
g: s__Streptococcus_lutetiensis
h: s__Staphylococcus_caprae
i: g__Staphylococcus
j: f__Staphylococcaceae
k: f__Acidothermaceae
l: s__Caulobacter_sp__g__Caulobacter
m: g__Caulobacter
o__Staphylococcales
o__Acetobacterales
s__Streptococcus_lutetiensis
g__Acidothermus
f__Acidothermaceae
s__unclassified_g__Acidothermus
g__Pedobacter
s__unclassified_g__Pedobacter
o__Acetobacterales
f__Acetobacteraceae
s__Staphylococcus_caprae
o__Staphylococcales
g__Staphylococcus
f__Staphylococcaceae
f__Weeksellaceae
s__Caulobacter_sp__g__Caulobacter
g__Caulobacter
LDA SCORE (log 10)

Figure S10. Bacterial taxa enriched in HBV-negative HCCs. A. The bacterial taxa showed a significant difference between HBV-negative (n=22) HccM and HBV-positive (n=49) HccM. The two-tailed p-value was calculated using Wilcoxon sum-rank test, and the p-value was adjusted using the FDR method. The 0.95% confidence intervals (CIs) were calculated using the bootstrap method. **B**. The cladogram and bar plot of LEfSe analysis showed the major taxa with the most abundance and a significant LDA score >2.5 in HBV-negative (n=22) HccM and HBV-positive (n=49) HccM.

Figure S11

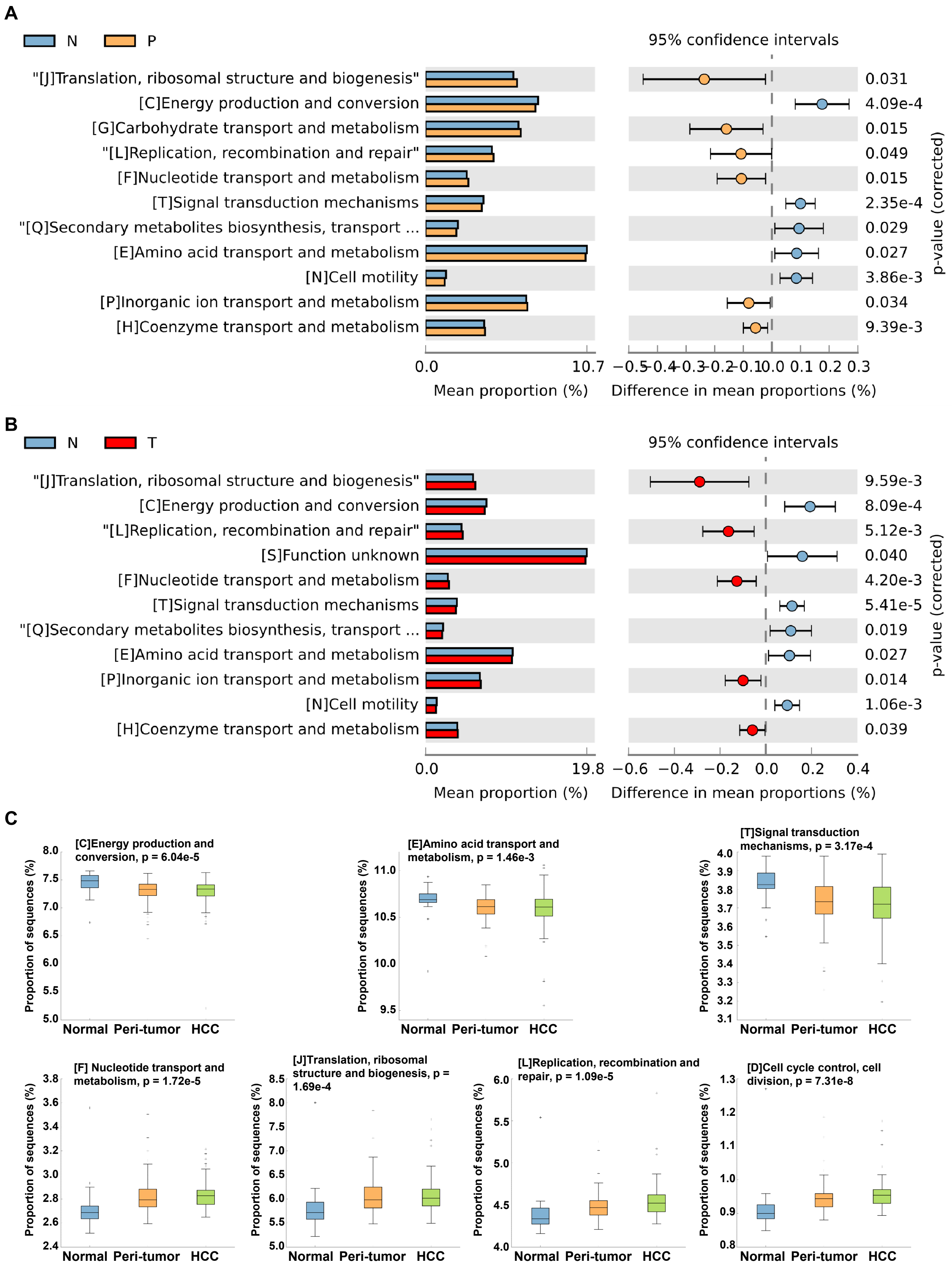

A
N
P
95% confidence intervals
"[J]Translation, ribosomal structure and biogenesis"
[C]Energy production and conversion
[G]Carbohydrate transport and metabolism
"[L]Replication, recombination and repair"
[F]Nucleotide transport and metabolism
[T]Signal transduction mechanisms
"[Q]Secondary metabolites biosynthesis, transport ...
[E]Amino acid transport and metabolism
[N]Cell motility
[P]Inorganic ion transport and metabolism
[H]Coenzyme transport and metabolism
0.031
4.09e-4
0.015
0.049
0.015
2.35e-4
0.029
0.027
3.86e-3
0.034
9.39e-3
p-value (corrected)
Mean proportion (%)
Difference in mean proportions (%)
B
N
T
95% confidence intervals
"[J]Translation, ribosomal structure and biogenesis"
[C]Energy production and conversion
"[L]Replication, recombination and repair"
[S]Function unknown
[F]Nucleotide transport and metabolism
[T]Signal transduction mechanisms
"[Q]Secondary metabolites biosynthesis, transport ...
[E]Amino acid transport and metabolism
[P]Inorganic ion transport and metabolism
[N]Cell motility
[H]Coenzyme transport and metabolism
9.59e-3
8.09e-4
5.12e-3
0.040
4.20e-3
5.41e-5
0.019
0.027
0.014
1.06e-3
0.039
p-value (corrected)
Mean proportion (%)
Difference in mean proportions (%)
C
[C]Energy production and conversion, p = 6.04e-5
[E]Amino acid transport and metabolism, p = 1.46e-3
[T]Signal transduction mechanisms, p = 3.17e-4
[F] Nucleotide transport and metabolism, p = 1.72e-5
[J]Translation, ribosomal structure and biogenesis, p = 1.69e-4
[L]Replication, recombination and repair, p = 1.09e-5
[D]Cell cycle control, cell division, p = 7.31e-8
Proportion of sequences (%)
Normal Peri-tumor HCC

Figure S11. Inferred COG function by PICRUSt across different liver microbiota. (**A**) and (**B**) Comparison of the abundance of COG categories between the normal and peri-tumor microbiota or between the normal and HCC microbiota. The significance was calculated using a two-sided Welch's t-test and the 95% confidence interval of the difference in mean proportion was calculated using Welch's inverted method. (**C**). The comparison of the abundance of selected COG categories in the normal, peri-tumor, and HCC microbiota. The comparison of function categories among the three groups was evaluated using the Kruskal–Wallis test followed by a post hoc Tukey-Kramer test (threshold = 0.95).

Figure S12

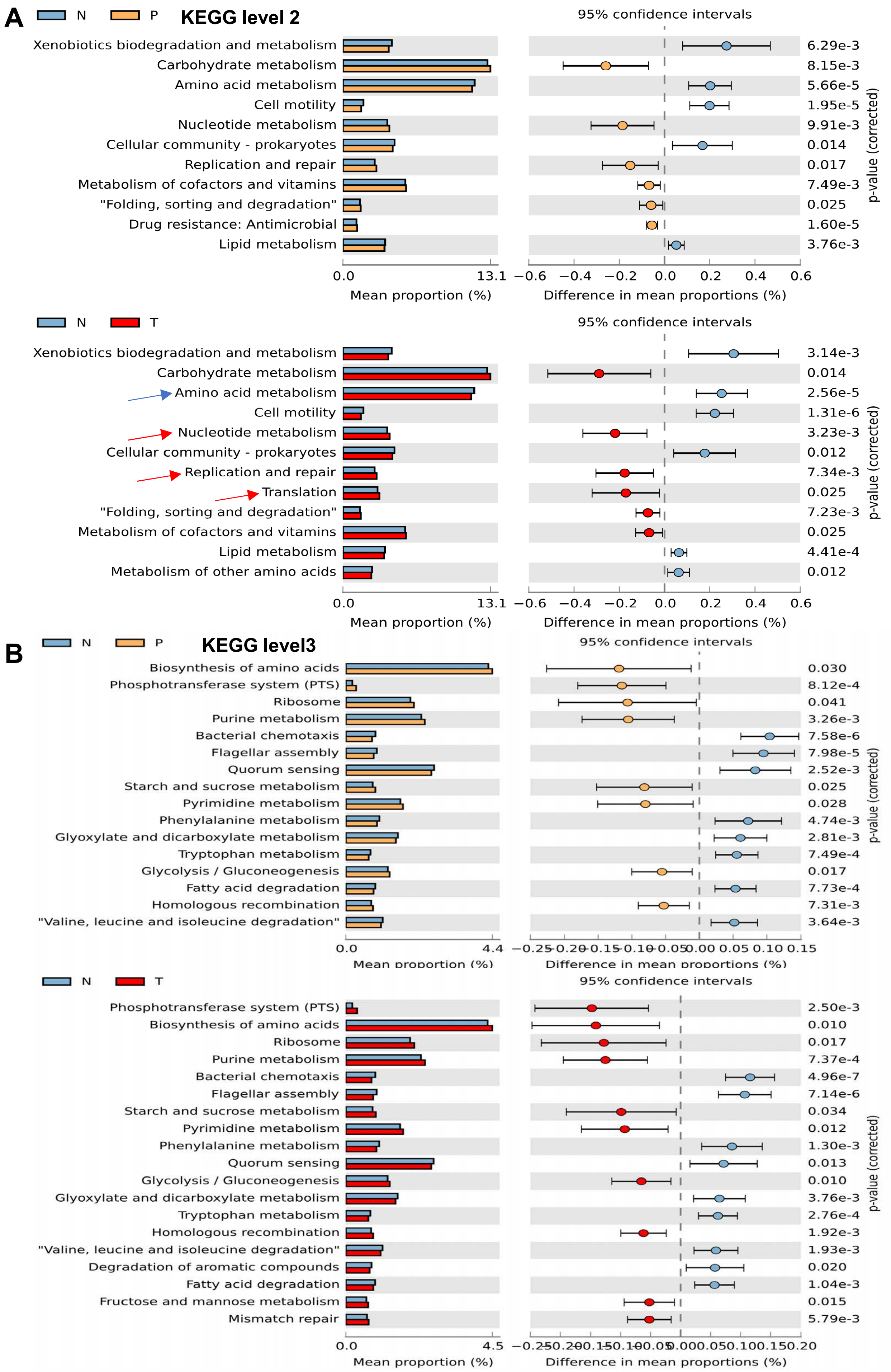
A
N
P
KEGG level 2
95% confidence intervals
Xenobiotics biodegradation and metabolism
Carbohydrate metabolism
Amino acid metabolism
Cell motility
Nucleotide metabolism
Cellular community - prokaryotes
Replication and repair
Metabolism of cofactors and vitamins
"Folding, sorting and degradation"
Drug resistance: Antimicrobial
Lipid metabolism
6.29e-3
8.15e-3
5.66e-5
1.95e-5
9.91e-3
0.014
0.017
7.49e-3
0.025
1.60e-5
3.76e-3
p-value (corrected)
0.0
13.1
−0.6 −0.4 −0.2 0.0 0.2 0.4 0.6
Mean proportion (%)
Difference in mean proportions (%)
N
T
95% confidence intervals
Xenobiotics biodegradation and metabolism
Carbohydrate metabolism
Amino acid metabolism
Cell motility
Nucleotide metabolism
Cellular community - prokaryotes
Replication and repair
Translation
"Folding, sorting and degradation"
Metabolism of cofactors and vitamins
Lipid metabolism
Metabolism of other amino acids
3.14e-3
0.014
2.56e-5
1.31e-6
3.23e-3
0.012
7.34e-3
0.025
7.23e-3
0.025
4.41e-4
0.012
p-value (corrected)
0.0
13.1
−0.6 −0.4 −0.2 0.0 0.2 0.4 0.6
Mean proportion (%)
Difference in mean proportions (%)
B
N
P
KEGG level3
95% confidence intervals
Biosynthesis of amino acids
Phosphotransferase system (PTS)
Ribosome
Purine metabolism
Bacterial chemotaxis
Flagellar assembly
Quorum sensing
Starch and sucrose metabolism
Pyrimidine metabolism
Phenylalanine metabolism
Glyoxylate and dicarboxylate metabolism
Tryptophan metabolism
Glycolysis / Gluconeogenesis
Fatty acid degradation
Homologous recombination
"Valine, leucine and isoleucine degradation"
0.030
8.12e-4
0.041
3.26e-3
7.58e-6
7.98e-5
2.52e-3
0.025
0.028
4.74e-3
2.81e-3
7.49e-4
0.017
7.73e-4
7.31e-3
3.64e-3
p-value (corrected)
0.0
4.4
Mean proportion (%)
Difference in mean proportions (%)
N
T
95% confidence intervals
Phosphotransferase system (PTS)
Biosynthesis of amino acids
Ribosome
Purine metabolism
Bacterial chemotaxis
Flagellar assembly
Starch and sucrose metabolism
Pyrimidine metabolism
Phenylalanine metabolism
Quorum sensing
Glycolysis / Gluconeogenesis
Glyoxylate and dicarboxylate metabolism
Tryptophan metabolism
Homologous recombination
"Valine, leucine and isoleucine degradation"
Degradation of aromatic compounds
Fatty acid degradation
Fructose and mannose metabolism
Mismatch repair
2.50e-3
0.010
0.017
7.37e-4
4.96e-7
7.14e-6
0.034
0.012
1.30e-3
0.013
0.010
3.76e-3
2.76e-4
1.92e-3
1.93e-3
0.020
1.04e-3
0.015
5.79e-3
p-value (corrected)
0.0
4.5
Mean proportion (%)
Difference in mean proportions (%)

Figure S12. Inferred KEGG function by PICRUSt across different liver microbiota. (**A**) Comparisons of the abundance of KEGG level2 categories between the normal and peri-tumor microbiota or between the normal and HCC microbiota. (**B**) Comparisons of the abundance of KEGG level3 categories between the normal and peri-tumor microbiota or between the normal and HCC microbiota.

Figure S13

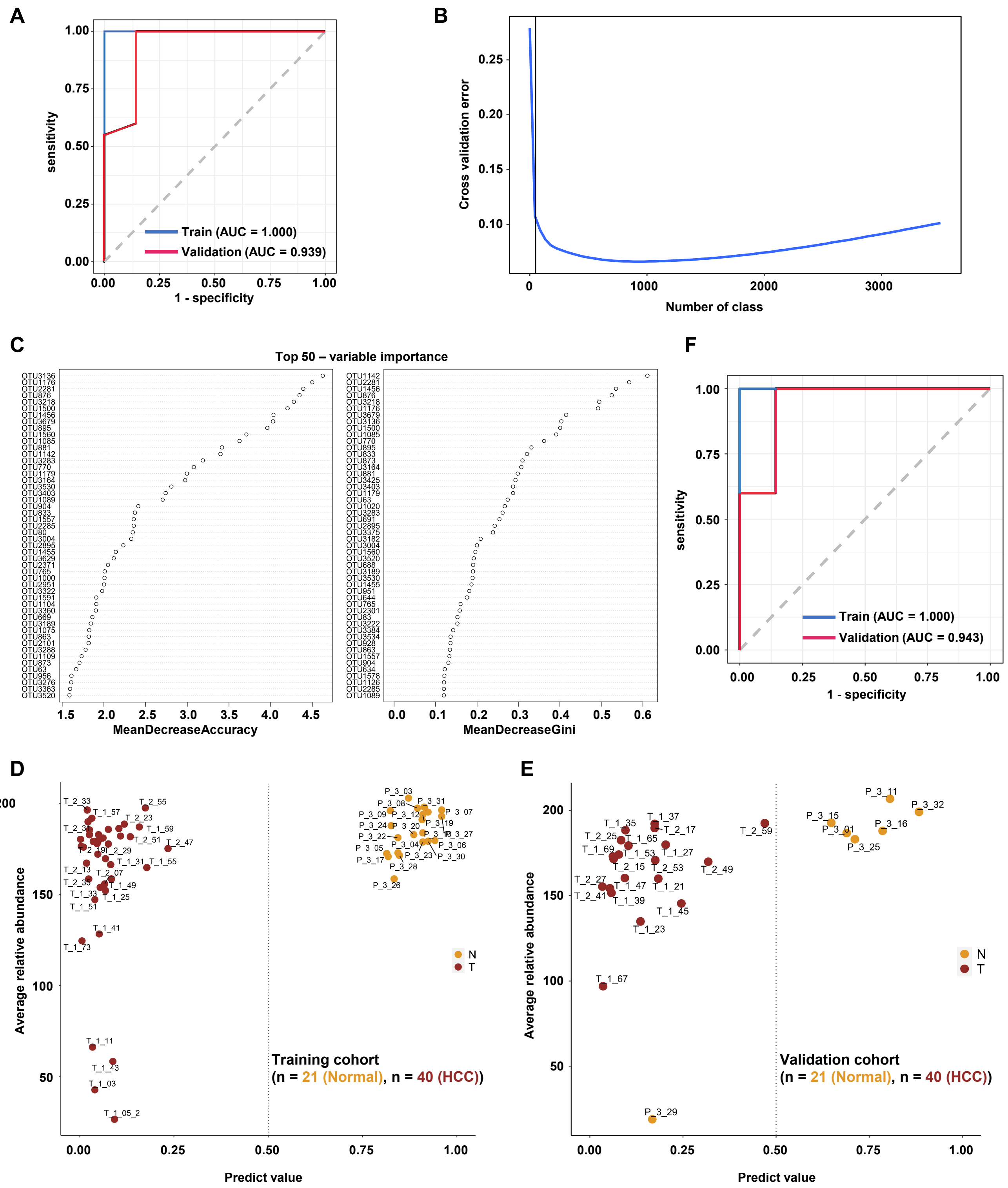
A
sensitivity
1 - specificity
Train (AUC = 1.000)
Validation (AUC = 0.939)
B
Cross validation error
Number of class
C
Top 50 – variable importance
MeanDecreaseAccuracy
MeanDecreaseGini
F
sensitivity
1 - specificity
Train (AUC = 1.000)
Validation (AUC = 0.943)
D
Average relative abundance
Predict value
Training cohort
(n = 21 (Normal), n = 40 (HCC))
E
Average relative abundance
Predict value
Validation cohort
(n = 21 (Normal), n = 40 (HCC))

Figure S13. Microbial OTU-based diagnostic biomarker for HCC. (**A**) Receiver operating characteristic (ROC) curves of prediction performance for the OTU-based predictors of the RF model built on all OTUs. The blue and red curves indicated the performance of the model in the training cohort (HCC patient = 40, healthy = 20) and validation cohort (HCC patient = 20, healthy = 7), respectively. (**B**) The cross-validation error curve shows the 10-cross validation method to prioritize the top features used to build a simplified model. (**C**) The top 50 OTUs are prioritized by the 10-cross validation method. (**D**) Performance of the simplified model using the top 50 OTUs in the training cohort containing 40 HCC patients and 20 healthy individuals. (**E**) Performance of the simplified model using the top 50 OTUs in the validation cohort containing 20 HCC patients and 7 healthy individuals. (**F**) ROC curves of prediction performance for the OTU-based predictors of the simplified RF model built on top-50 OTUs. The blue and red curves indicated the performance of the model in the training cohort (HCC patient = 40, healthy = 20) and validation cohort (HCC patient = 20, healthy = 7), respectively.

References

1. Nejman D, Livyatan I, Fuks G, Gavert N, Zwang Y, Geller LT, Rotter-Maskowitz A, Weiser R, Mallel G, Gigi E, Meltser A, Douglas GM, Kamer I, Gopalakrishnan V, Dadosh T, Levin-Zaidman S, Avnet S, Atlan T, Cooper ZA, Arora R, Cogdill AP, Khan MAW, Ologun G, Bussi Y, Weinberger A, Lotan-Pompan M, Golani O, Perry G, Rokah M, Bahar-Shany K, Rozeman EA, Blank CU, Ronai A, Shaoul R, Amit A, Dorfman T, Kremer R, Cohen ZR, Harnof S, Siegal T, Yehuda-Shnaidman E, Gal-Yam EN, Shapira H, Baldini N, Langille MGI, Ben-Nun A, Kaufman B, Nissan A, Golan T, Dadiani M, et al. 2020. The human tumor microbiome is composed of tumor type-specific intracellular bacteria. Science 368:973-980.
2. Pfeiffer S, Pastar M, Mitter B, Lippert K, Hackl E, Lojan P, Oswald A, Sessitsch A. 2014. Improved group-specific primers based on the full SILVA 16S rRNA gene reference database. Environ Microbiol 16:2389-407.
3. Sookoian S, Salatino A, Castano GO, Landa MS, Fijalkowky C, Garaycoechea M, Pirola CJ. 2020. Intrahepatic bacterial metataxonomic signature in non-alcoholic fatty liver disease. Gut 69:1483-1491.
